# SFINN: inferring gene regulatory network from single-cell and spatial transcriptomic data with shared factor neighborhood and integrated neural network

**DOI:** 10.1093/bioinformatics/btae433

**Published:** 2024-07-01

**Authors:** Yongjie Wang, Fengfan Zhou, Jinting Guan

**Affiliations:** Department of Automation, Xiamen University, Xiamen, Fujian 361102, China; Department of Automation, Xiamen University, Xiamen, Fujian 361102, China; Department of Automation, Xiamen University, Xiamen, Fujian 361102, China; Key Laboratory of System Control and Information Processing, Ministry of Education, Shanghai 200240, China; National Institute for Data Science in Health and Medicine, Xiamen University, Xiamen, Fujian 361102, China

## Abstract

**Motivation:**

The rise of single-cell RNA sequencing (scRNA-seq) technology presents new opportunities for constructing detailed cell type-specific gene regulatory networks (GRNs) to study cell heterogeneity. However, challenges caused by noises, technical errors, and dropout phenomena in scRNA-seq data pose significant obstacles to GRN inference, making the design of accurate GRN inference algorithms still essential. The recent growth of both single-cell and spatial transcriptomic sequencing data enables the development of supervised deep learning methods to infer GRNs on these diverse single-cell datasets.

**Results:**

In this study, we introduce a novel deep learning framework based on shared factor neighborhood and integrated neural network (SFINN) for inferring potential interactions and causalities between transcription factors and target genes from single-cell and spatial transcriptomic data. SFINN utilizes shared factor neighborhood to construct cellular neighborhood network based on gene expression data and additionally integrates cellular network generated from spatial location information. Subsequently, the cell adjacency matrix and gene pair expression are fed into an integrated neural network framework consisting of a graph convolutional neural network and a fully-connected neural network to determine whether the genes interact. Performance evaluation in the tasks of gene interaction and causality prediction against the existing GRN reconstruction algorithms demonstrates the usability and competitiveness of SFINN across different kinds of data. SFINN can be applied to infer GRNs from conventional single-cell sequencing data and spatial transcriptomic data.

**Availability and implementation:**

SFINN can be accessed at GitHub: https://github.com/JGuan-lab/SFINN.

## 1 Introduction

Gene regulatory network (GRN) describes the interactions and regulatory relationships between transcription factors (TFs) and their targets genes (Luo and Woolf 2010). The regulatory interactions are influenced by cell’s epigenetic state, dependent upon TF binding activity, histone modification, and chromatin accessibility, which have been associated with cell type-specific expression (Siahpirani *et al.* 2022). Cells harbor identical genomes but behave differently, which is due to that GRNs reconfigure during dynamic processes, such as development or disease progression, to specify cell type-specific expression levels (Zhang *et al.* 2023). Therefore, accurate inference of cell type-specific GRNs can reveal key regulatory factors and circuits for specific cell types, which is crucial for understanding the regulatory programs for the differentiation and maintenance of distinct cellular states and studying various biological processes and diseases (Cha and Lee 2020).

In the past few decades, numerous methods have been developed for inferring relationships between genes (Faith *et al.* 2007, Huynh-Thu *et al.* 2010, Krishnaswamy *et al.* 2014, Chan *et al.* 2017, Jafari *et al.* 2017, Moerman *et al.* 2019). These methods can be broadly categorized into two types: traditional statistical and machine learning-based approaches, and deep learning-based approaches (Mochida *et al.* 2018). Among them, the traditional statistical methods include algorithms based on correlation and information entropy. Algorithms such as those using Pearson correlation coefficient (PCC), mutual information (MI), or conditional mutual information are commonly used to study the relationships between genes. PCC is simple, intuitive, and computationally efficient but may exhibit a weak response to nonlinear gene regulatory relationships, potentially failing to comprehensively reflect true biological regulatory connections. MI, on the other hand, possesses the ability to capture both linear and nonlinear relationships between genes but can be prone to confusion between direct and indirect regulatory relationships (Delgado and Gómez-Vela 2019). DREMI is an entropy-based algorithm that utilizes the kernel density of heat diffusion to infer joint density, followed by the estimation of conditional density to infer the information transfer between genes (McCalla *et al.* 2023). Compared with MI, DREMI is better at capturing the gene correlation, but its performance is significantly influenced by the choice of the density estimator. Knn-DREMI, an improved algorithm based on DREMI, addresses this limitation by employing a *K* nearest neighbors-based density estimator, enabling better adaptation to the high-dimensional and sparse natures of single-cell RNA-seq data (van Dijk *et al.* 2018). Among the traditional machine learning-based algorithms, GENIE3 is a representative, which integrates multiple regression tree models to infer relationships between genes, and has demonstrated excellent performance in DREAM (Dialogue for Reverse Engineering Assessments and Methods) network inference challenges and recent benchmark tests on real gene expression data (Pratapa *et al.* 2020, Kang *et al.* 2021). Though these methods, employing diverse mathematical and statistical principles, provide a systematic framework for studying gene relationships within cellular systems, many of them were developed originally for bulk gene expression data.

With the development of high-throughput single-cell transcriptomic sequencing technologies, there is a pressing need for a more flexible and powerful computational approach that can effectively extract essential information from the rapidly accumulating and increasingly complex biological data. Recently, deep learning-based methods have been developed to autonomously learn features and patterns for better understanding and interpreting the relationships between genes. CNNC (Yuan and Bar-Joseph 2019) is a supervised deep learning framework based on convolutional neural networks (CNNs). It encodes the gene pair expression into 2D histograms, employs the architecture of CNNs to handle co-expression patterns of gene pairs, and infers potential regulatory relationships among genes. Additionally, in the article of CNNC, a deep learning framework called DNN was introduced based on multiple fully-connected layers to predict interactions between genes, which also requires the conversion of joint expression of gene pair into histogram images. SDINet (Li *et al.* 2022) is a supervised method that proposes the fusion of two modalities by applying a novel CNN to extract gene regulatory interactions from gene expression images and RNA-seq data. Similarly, SDINet requires the conversion of RNA-seq data into histogram form. What’s more, single-cell spatial transcriptomics data, which reflects the expression and spatial information of single cells, provide researchers with the opportunity to integrate the location for exploring GRNs in depth (Svensson *et al.* 2018, Littman *et al.* 2023). Among the analytical tools applicable to spatial transcriptomic data for GRN inference, the representatives are Giotto (Dries *et al.* 2021) and GCNG (Yuan and Bar-Joseph 2020). Giotto uses information about the location of cells in space to calculate ligand–receptor-based co-expression scores to model interaction strengths (Wang *et al.* 2023). GCNG does this by encoding the spatial information into a cell neighborhood map, then running the cell neighborhood map and the ligand–receptor gene expression data through a graph convolutional neural network (GCN), and ultimately outputting a dichotomous value to represent whether or not the ligand–receptor interactions are present in that map.

Although the existing GRN methods facilitate the identification of gene relationships, many of them were not developed specifically for single-cell gene expression data, not being able to simultaneously incorporate spatial location, and could not differentiate gene interactions and causalities. The increase of single-cell and spatial transcriptomic data drives us to design a supervised deep learning framework for the effective learning of feature representation of gene pair expression and integration of cell spatial location information. To this end, we develop a deep learning framework called SFINN based on shared factor neighborhood (SFN) and integrated neural network, which can be applied on single-cell gene expression data and spatial transcriptomic data for gene interaction and causality identification. For single-cell gene expression data, our approach generates a cell neighborhood network based on gene expression using SFN strategy. For spatial transcriptomic datasets, we integrate the cell network derived from gene expression matrix with that derived using spatial positional information. Moreover, we introduce an integrative neural network framework comprising a GCN and a fully-connected neural network with the goal of identifying regulatory relationships between genes. In the framework of SFINN, gene pair expression data and cell neighborhood network serve as inputs to the GCN, while gene pair expression data are used independently as input to the fully-connected neural network. We evaluate the performance of SFINN in the tasks of inferring pairwise gene interactions and causal relationships using multiple single-cell transcriptomic datasets and spatial transcriptomic datasets against the existing GRN algorithms. Experimental results demonstrate that SFINN exhibits competitiveness and robustness when handling different types of gene expression datasets.

## 2 Materials and methods

### 2.1 Datasets

#### 2.1.1 Single-cell transcriptomic datasets

We used eight single-cell transcriptomic datasets to assess the ability of SFINN in identifying TF–target gene interactions and also causal relationships. We obtained three single-cell transcriptomic datasets along with their corresponding ground-truth (ChIP-Seq data) from the study of Yuan and Bar-Joseph (2019), including datasets of bone marrow-derived macrophages (Alavi *et al.* 2018), dendritic single cells (Alavi *et al.* 2018), and IB10 mouse embryonic stem cells (mesc) (Klein *et al.* 2015). For the selection of TFs, we adhered to the previous practice (Yuan and Bar-Joseph 2019). Additionally, we collected five single-cell transcriptomic datasets and their associated ground-truth ChIP-Seq data from the study conducted by Chen *et al.* (2021), including datasets of 5G6GR mouse embryonic stem cells [mESC (2)] (Hayashi *et al.* 2018), human embryonic stem cells (hESC) (Chu *et al.* 2016), and the three lineages of mouse hematopoietic stem cells (mHSC) including mHSC-E, mHSC-GM, and mHSC-L (Nestorowa *et al.* 2016). Regarding these datasets, we randomly selected 18 TFs from the overall ground-truth set like the way in the previous study (Chen *et al.* 2021). In the task of predicting gene interaction, we processed the ground-truth data to label the gene pair *a* and *b* as “1” if gene *a* interacts with gene *b*, otherwise as “0”. In the task of predicting causal relationship, we processed the ground-truth to label the gene pair *a* and *b* as “1” if gene *a* regulates gene *b*, otherwise as “0”. The specific details for each dataset are provided in Table 1 and Supplementary Material.

**Table 1.** Eight single-cell transcriptomic datasets and five spatial transcriptomic datasets used in the experiments.

Type of dataset	Dataset	Number of cells	Number of genes	Size of training set	Number of TFs
Single-cell transcriptomic dataset	Bone marrow-derived macrophages (Alavi *et al.* 2018)	6283	20 463	53 502	13
	Dendritic (Alavi *et al.* 2018)	4126	20 463	29 432	16
	mesc (Klein *et al.* 2015)	2717	24 175	174 582	38
	hESC (Chu *et al.* 2016)	758	17 735	100 720	18
	mESC(2) (Hayashi *et al.* 2018)	421	18 835	94 332	18
	mHSC-E (Nestorowa *et al.* 2016)	1071	4762	49 114	18
	mHSC-GM (Nestorowa *et al.* 2016)	889	4762	43 712	18
	mHSC-L (Nestorowa *et al.* 2016)	847	4762	48 884	18
Spatial transcriptomic datasets	seqFISH+ (Eng *et al.* 2019)	913	10 000	2112	286
	MERFISH (Xia *et al.* 2019)	1368	10 050	1682	235
	ST_SCC_P2_1 (Ji *et al.* 2020)	666	17 138	3728	520
	ST_SCC_P2_2 (Ji *et al.* 2020)	646	17 344	3792	526
	ST_SCC_P2_3 (Ji *et al.* 2020)	638	17 833	3878	543

#### 2.1.2 Single-cell spatial transcriptomic datasets

For the tasks of inferring gene interactions and causal relationships, we analysed five single-cell spatial transcriptomic datasets (Table 1 and Supplementary Material), including seqFISH+ dataset (Eng *et al.* 2019), MERFISH dataset (Xia *et al.* 2019), and the datasets of three cryosections from a patient with squamous cell carcinoma (denoted as ST_SCC_P2_1, ST_SCC_P2_2, and ST_SCC_P2_3) (Ji *et al.* 2020). We downloaded the list of real interacting ligand–receptor from Yuan and Bar-Joseph (2020). For the interaction prediction task, if both genes of an interacting gene pair exist in the gene expression matrix, we retained the gene pair as a positive gene pair. The genes that are not in the receptor list but present in the gene expression matrix were selected as the target genes for the non-interacting gene pairs, keeping the ratio of the numbers of positive and negative gene pairs as 1:1. For the causality prediction task, we randomly selected positive pairs from the true list of ligand–receptor interactions and generated negative pairs for it. Specifically, for each known ligand–receptor gene pair *a* and *b* with label of “1”, a negative pair *b* and *a* with label of “0” was introduced. Normalization procedures for raw spatial gene expression data followed the steps of Eng *et al.* (2019) and Yuan and Bar-Joseph (2020).

### 2.2 Training and testing strategy

For the TF–target gene interaction and causality prediction tasks on both single-cell transcriptomic datasets and single-cell spatial transcriptomic datasets, we employed 3-fold cross-validation method for evaluation. We divided the total TFs into three parts, with two parts of TFs and their target genes used for model training and the remaining one used for testing. This strictly ensures that there is no overlap of TF–target gene pairs between the entire training dataset and the testing dataset, thus avoiding information leakage. For the interaction prediction task, to ensure that the positive and negative gene pairs of each TF are balanced, we randomly selected an equal number of TF–nontarget gene pairs as negative examples for positive gene pairs, that is, for each positive TF–target gene pair (*a*, *x*1), there is a negative TF–nontarget pair (*a*, *x*2). As to the causality prediction task, for the positive gene pair (*a*, *x*1), we introduced the negative gene pair (*x*1, *a*).

### 2.3 Construction of cell–cell adjacency matrix

For single-cell transcriptomic data, we firstly obtained a low-dimensional matrix *H* by performing principal component analysis (PCA) on the gene expression matrix $X_{c,g}$ (where *c* is the number of cells and *g* is the number of genes in the training set), making the cumulative variance contribution rate of the principal components reaching 90%. Next, SFN strategy (Welch *et al.* 2019) was used to compute the distance between cells. Specifically, based on *H*, identify the *K* neighbors for each sample. In our approach, *K* is set to a default value of 10. Then, find the factor with the highest expression in sample *i*, and mark it as *f*(*i*). Collect the highest factors of *K* neighbors for sample *i*, resulting in a histogram vector FN(*i*). Next, calculate the Manhattan distance matrix *E* whose entry $E\left(i,j\right)$ is calculated using the histogram vectors of samples *i* and *j*. Finally, calculate the similarity matrix whose entry is computed as:

(1) $$\mathrm{similarity}\;\mathrm{matrix}\left(i,j\right)=\frac{1}{1+E\left(i,j\right)}$$

Subsequently, a threshold (by default 0.5) was applied to determine a cell–cell adjacency matrix. When the similarity value is greater than the threshold, we set the corresponding adjacency matrix element to one; otherwise, it is set to zero.

For single-cell spatial transcriptomic datasets, as they provide cellular spatial location data, we initially calculated the Euclidean distance based on the spatial positional information and then converted it into a similarity matrix like Equation (1). After applying a threshold, we also transformed the similarity matrix to a cell–cell adjacency matrix. To integrate the two cell–cell adjacency matrices derived from gene expression and spatial location into one final cell–cell adjacency matrix, we performed a logical OR operation, i.e. when the corresponding elements at the same position in both matrices are zeros, we set the entry of the final cell–cell adjacency matrix to zero; otherwise, we set it to one.

### 2.4 SFINN framework

SFINN is a supervised neural network framework integrating two parallel components, namely a graph convolutional neural network (GCN) module and a fully-connected neural network (NN) module. For the NN module, gene pair expression is used as input. Since the numbers of cells in different single-cell transcriptomic datasets are different, and some datasets have a small number of cells, we designed the neural network as a shallow structure to avoid overfitting. The NN contains two dense layers, each containing 32 neurons, and the mathematical formula for the propagation is defined as:

(2) $$X^{\left(l+1\right)}=\rho \left(\mathrm{linear}\left(X^{\left(l\right)}\right)\right)$$

where *ρ* represents the nonlinear activation function Relu used for the output of each layer, $X^{\left(l\right)}$ represents the input of the *l*th layer, and $X^{\left(0\right)}$ is the original input of gene pair expression value.

For the GCN module, the expression values of gene pair and the cell–cell adjacency matrix are inputs. The GCN module consists of two graph convolutional layers, and the specific propagation formula is defined as:

(3) $$X^{\left(l+1\right)}=\sigma \left(D^{-\frac{1}{2}}AD^{\frac{1}{2}}X^{\left(l\right)}W^{\left(l\right)}+b^{\left(l\right)}\right)$$

Here, $X^{\left(l+1\right)}$ represents the embedded representation learned in the (*l *+* *1)th layer, *σ* denotes the nonlinear activation function Elu used in the graph convolutional layers, *A* is the cellular neighborhood network, *D* is the degree matrix of *A*, $X^{\left(l\right)}$ represents the output feature embedding of the previous GCN layer, and $X^{0}$ corresponds to the original input of gene pair expression values. $W^{\left(l\right)}$ and $b^{\left(l\right)}$, respectively represent the weight matrix and bias term of the *l*th layer.

For the features learned by GCN and NN, we denote them as $\theta_{1}$ and $\theta_{2}$, respectively. These features are concatenated using a concatenate layer, followed by a flatten layer, and then a dense layer. Finally, the output layer uses a sigmoid function for classification, producing the regulatory probability of the input gene pair. SFINN transforms the task of constructing GRNs into a binary classification problem, with binary cross-entropy loss function serving as the objective optimization function:

L=-∑i=1Tyilog ⁡SFINNΘx

(4) $$+\left(1-y_{i}\right)\sum_{i=1}^{T}y_{i}\left(1-\log\left(1-{\mathrm{SFINN}}_{\Theta }\left(x\right)\right)\right)$$

Here, *i* represents the *i*th gene pair, $y_{i}$ denotes the label for the *i*th pair, *T* is the total number of gene pairs, *Θ* represents all the parameters in the SFINN model.

### 2.5 Benchmarking

When testing on conventional single-cell datasets, we compared SFINN against seven different GRN algorithms. These include methods based on statistics and traditional machine learning, encompassing PC (Jafari *et al.* 2017), MI (Faith *et al.* 2007), DREMI (Krishnaswamy *et al.* 2014), PIDC (Chan *et al.* 2017), and the regression-based GENIE3 (Huynh-Thu *et al.* 2010), and deep learning methods, DNN (Yuan and Bar-Joseph 2019) and CNNC (Yuan and Bar-Joseph 2019). For DREMI, we specifically opted for the Knn-DREMI (van Dijk *et al.* 2018) version tailored to scRNA-seq data. For testing on spatial transcriptomic datasets, we selected the top three performing methods on single-cell transcriptomic datasets, as well as Giotto (Dries *et al.* 2021) and GCNG (Yuan and Bar-Joseph 2020) specifically developed for spatial transcriptomic datasets, to compare with SFINN. All methods were tested using default hyperparameters. AUROC (area under the receiver operating characteristic curve) (Davis and Goadrich 2006) and AUPR (area under the precision–recall curve) (Hughes-Oliver 2018) were utilized as measures to assess the performance of various methods in inferring GRN capabilities. ROC curve is drawn by calculating the true positive rate and false positive rate at each different threshold, and PR curve is drawn by calculating the precision and recall at each different threshold. For this, we compared the inferred edge lists between genes with the ground-truth and the cutoff of correlation value was varied to calculate each point of ROC or PR curve. Generally, a higher AUROC or AUPR value indicates a higher accuracy of the classifier in predicting gene regulatory relationships.

## 3 Results

### 3.1 Description of the overall analysis

**Figure 1.**
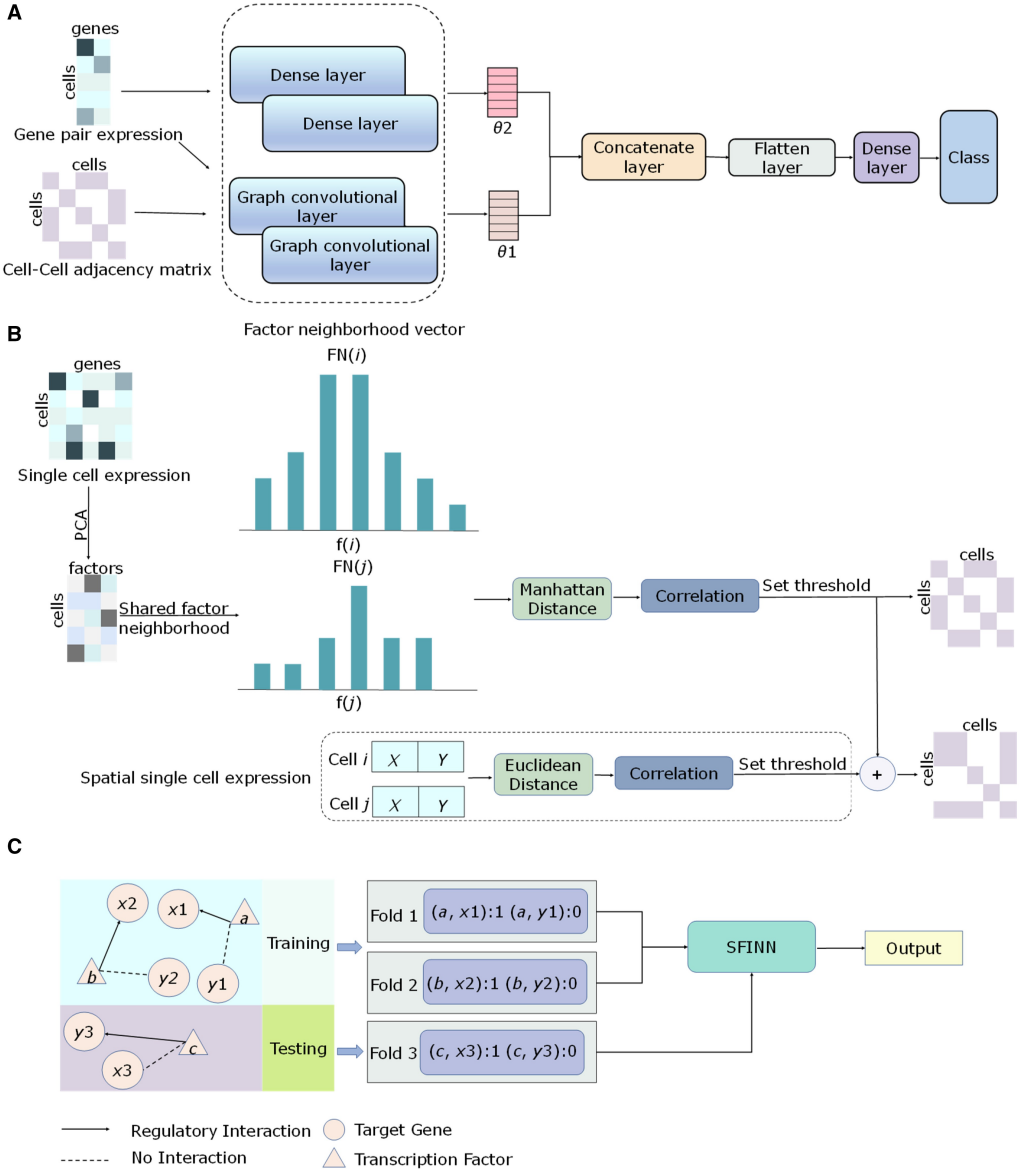
Overview of SFINN. (A) SFINN utilizes gene pair expression and cell–cell neighborhood graph as inputs for predicting if there is a regulatory relationship between the genes. (B) SFINN fuses the cell–cell adjacency matrix generated by shared factor neighborhood strategy and that generated using cell spatial location. (C) Training and testing data partitioning strategy. The existing ground-truth data are divided into three parts through cross-validation based on the number of TFs, ensuring a 1:1 ratio of positive to negative pairs. Additionally, genes in the testing dataset are strictly separated from genes in the training dataset.

We propose SFINN model based on SFN and integrated neural network to infer gene interactions and causalities from single-cell and spatial transcriptomic datasets. The entire analysis workflow is shown in Fig. 1. SFINN uses the gene pair expression data and the cellular neighborhood network as inputs to the GCN, while the gene pair expression is used as input to the fully-connected neural network. We fuze the feature extraction results from the two networks through the subsequent network layers and generate the final classification output (Fig. 1A). To construct the cell–cell neighborhood network, for single-cell transcriptomic dataset we performed PCA on gene expression data, constructed factor neighborhood vectors by SFN strategy to calculate the Manhattan distance of cells, and then transformed the distance to similarity for determining a cell–cell adjacency matrix. For spatial transcriptomic data, we calculated the Euclidean distance of cells using location information and converted it to a similarity matrix and then a cell–cell adjacency matrix. By performing a logical OR operation, we fused the two adjacency matrices (Fig. 1B). To train the model, we divided the gene pair dataset into a training set and a test set according to the number of TFs using 3-fold cross-validation, ensuring no overlap between the training and testing sets (Fig. 1C). AUPRC and AUROC scores were used as performance metrics to evaluate SFINN.

### 3.2 Gene interaction prediction on single-cell transcriptomic datasets

**Figure 2.**
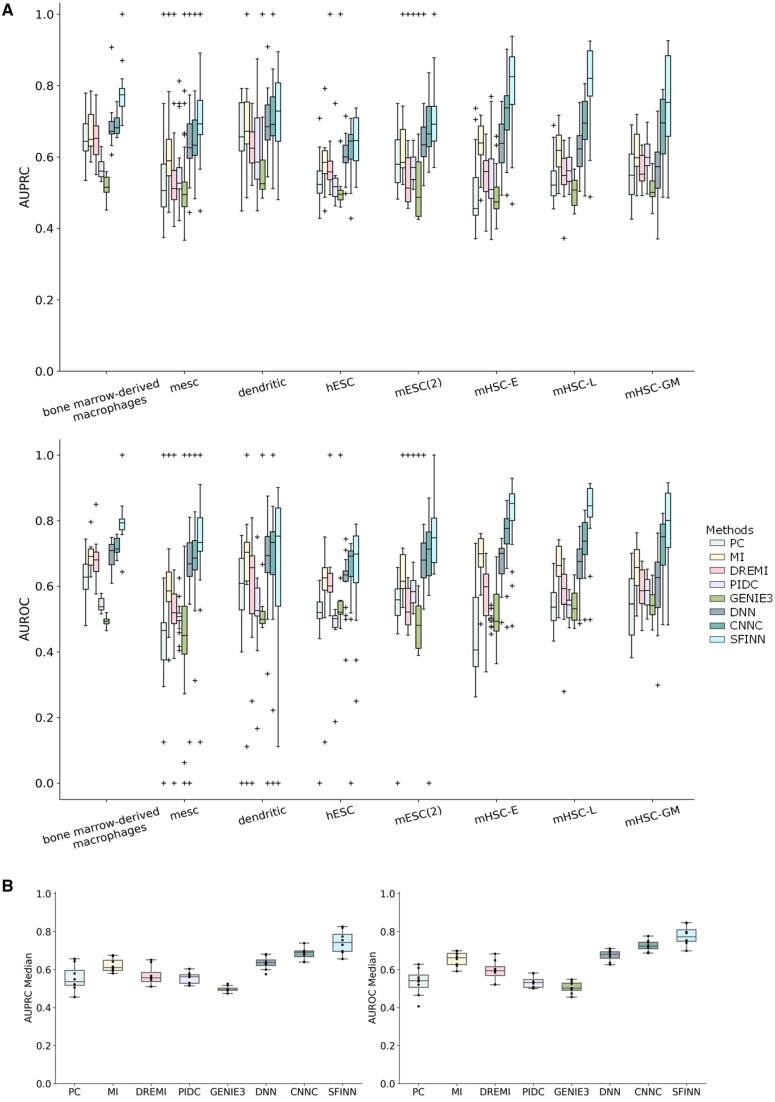
Performance comparison of SFINN with the existing methods in the task of gene interaction prediction across eight single-cell transcriptomic datasets. (A) The performance of SFINN and seven other GRN methods on eight datasets, each dot denoting the AUROC/AUPRC score for the gene pairs of each transcription factor. (B) The AUPRC/AUROC median across all transcription factors of each method on each dataset.

We used eight single-cell transcriptomic datasets to evaluate the performance of various methods in predicting cell type-specific gene interactions. Specifically, we compared our SFINN model with seven existing computational methods for GRN inference, including PC, MI, DREMI, GENIE3, PIDC, DNN, and CNNC. Among these, DNN and CNNC are supervised learning methods, while the remaining five are unsupervised learning methods. For each dataset, the corresponding ROC and PR curves of each method are shown as Supplementary Figs S1–S8. We calculated the AUROC/AUPRC score for the gene pairs of each TF, and listed the median (mean) across all TFs (Supplementary Figs S1–S8). From the figures, we can observe that SFINN performs well, followed by CNNC. For each dataset, we also pooled the AUROC/AUPRC scores of all TFs as Fig. 2A. It can be seen that SFINN performs the best overall across the eight datasets. In addition, we aggregated the median for each method on each dataset (Fig. 2B). It is evident that SFINN, CNNC, DNN, and MI are the top four methods in terms of overall performance. Supervised learning methods consistently outperformed unsupervised learning methods. The results indicate the effectiveness of SFINN in predicting TF–target gene interactions from single-cell transcriptomic datasets.

### 3.3 Gene interaction prediction on spatial transcriptomic datasets

**Figure 3.**
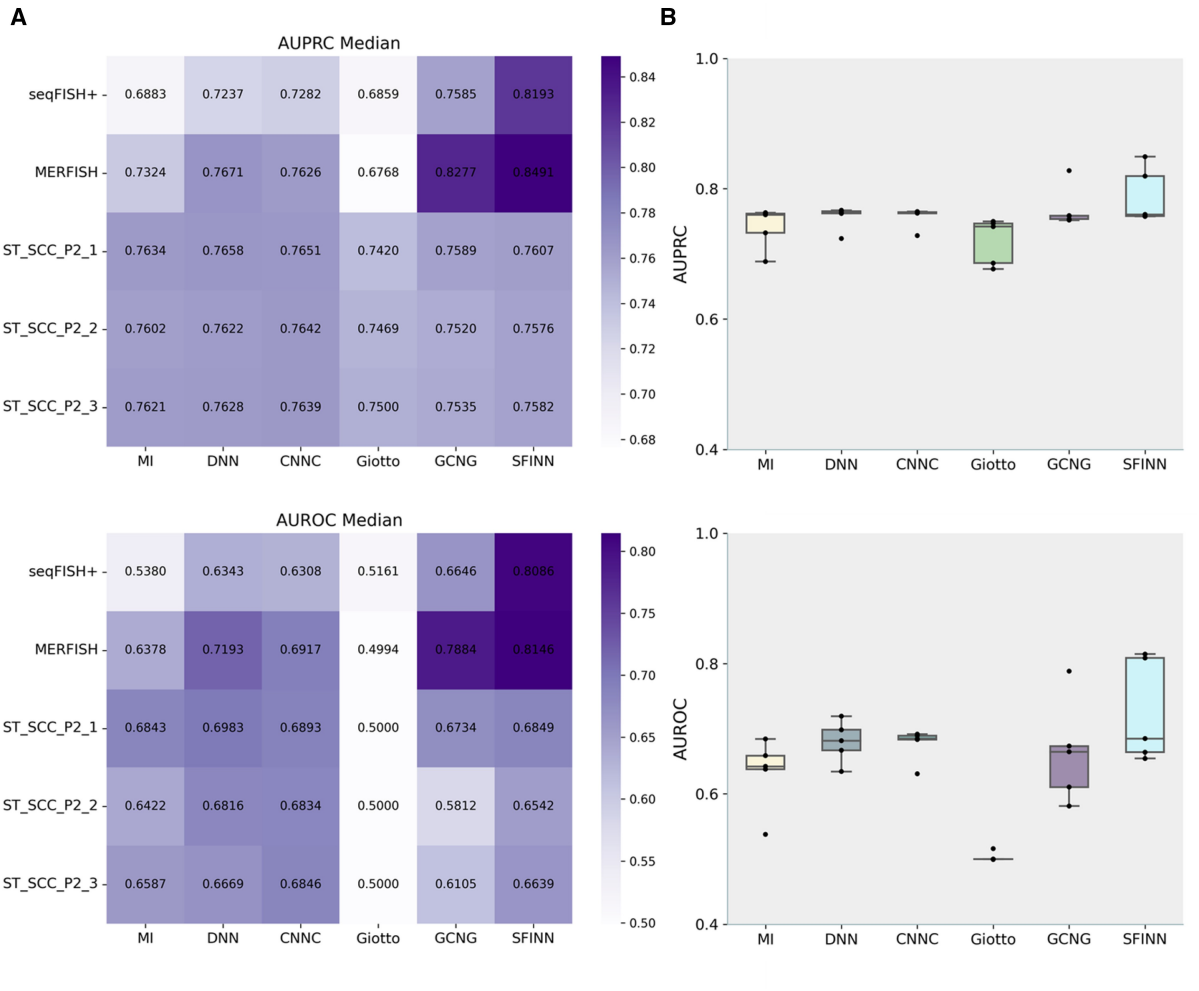
Performance comparison of SFINN with the existing algorithms in the task of gene interaction prediction across five spatial transcriptomic datasets. (A) The AUPRC/AUROC median across gene pairs of all transcription factors on each dataset for SFINN and the other five GRN methods. (B) The AUPRC/AUROC median on each dataset of each GRN method.

Based on the excellent performance of SFINN on single-cell transcriptomic datasets, we continued to test it on five single-cell spatial transcriptomic data. The top three methods in the task of inferring gene interactions from single-cell transcriptomic data in the first part, CNNC, DNN, and MI, as well as the methods specifically developed for spatial transcriptomic data, Giotto and GCNG, were selected to compare with SFINN. Figure 3A shows the AUROC/AUPRC median across gene pairs of all TFs for each method on each experimental dataset. The corresponding ROC and PR curves are shown in Supplementary Figs S9–S13. We can see that SFINN significantly outperforms the other methods on seqFISH+ and MERFISH, with AUPRC median being 6% and 2% higher than the second-best method, GCNG, and the median of AUROC being 15% and 3% higher than GCNG. In addition, SFINN is also competitive on the other three datasets. Figure 3B summarizes the median values of all datasets, showing the ability of SFINN in inferring gene interactions from spatial transcriptomic data. Meanwhile, some methods not specifically developed for single-cell spatial data, such as MI, DNN, and CNNC, also show good performance on these five spatial transcriptomic datasets, especially on the three datasets of ST_SCC_P2 series.

### 3.4 Gene causality prediction on single-cell and spatial transcriptomic datasets

**Figure 4.**
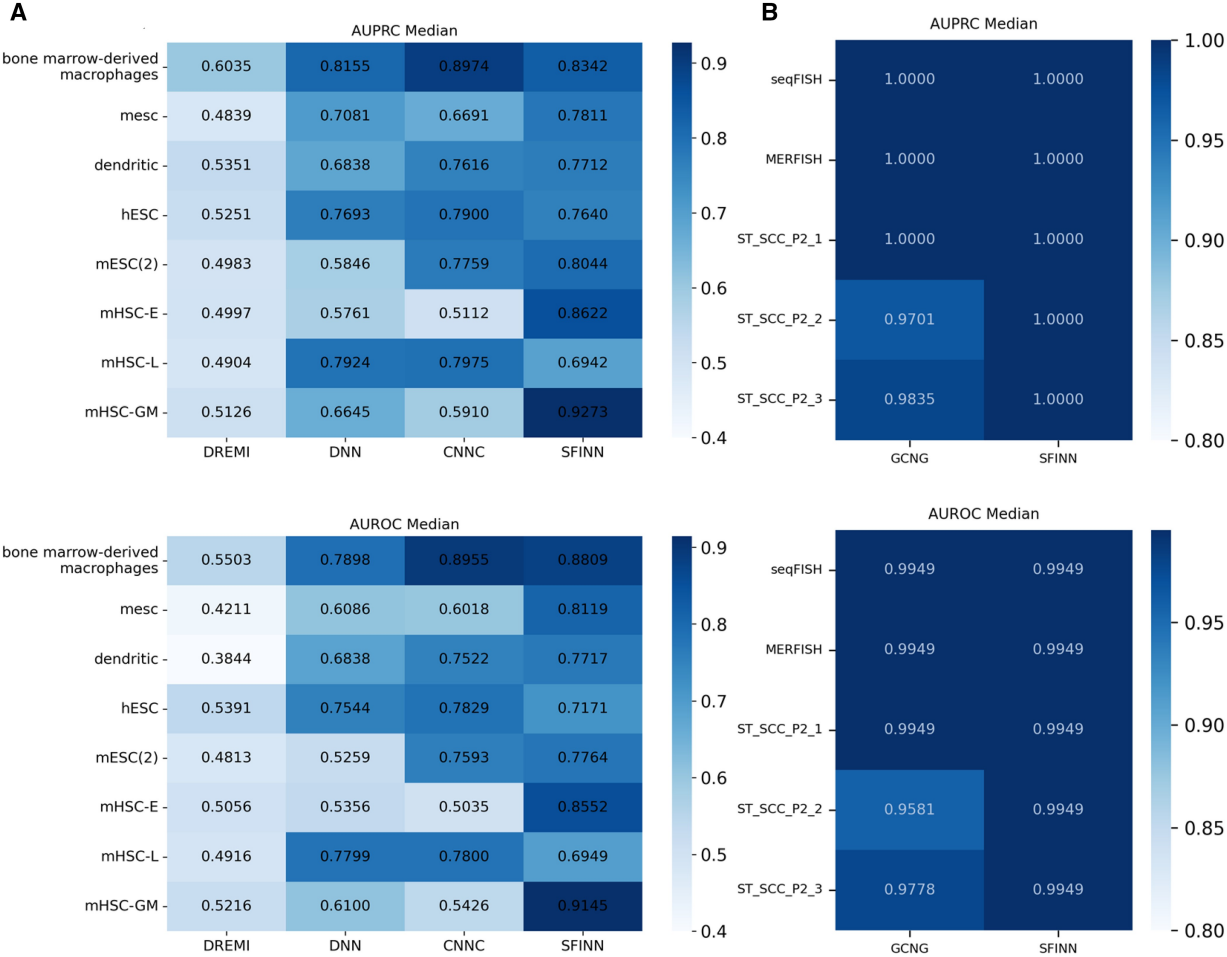
Performance comparison of SFINN with the other algorithms applicable for gene causality prediction. The AUPRC/AUROC median across gene pairs of all transcription factors on each dataset for SFINN and the other GRN methods inferring gene causality from (A) single-cell transcriptomic data and (B) spatial transcriptomic data.

Next, we validated the performance of SFINN in the task of causal relationship prediction. DREMI, DNN, and CNNC are suitable for causality inference task on single-cell expression data and GCNG is applicable for inferring gene causality on spatial transcriptomic data, therefore we compared SFINN with these methods. The median of AUROC/AUPRC of each method on each single-cell gene expression data is shown in Fig. 4A. SFINN obtains the highest AUPRC/AUROC scores on the majority of datasets. For all five spatial transcriptomic datasets, SFINN performs satisfactorily and competitive with GCNG (Fig. 4B). GCNG performs similarly to SFINN on seqFISH, MERFISH, and ST_SCCC_P2_1 datasets, but is slightly inferior to SFINN on the other two spatial transcriptomic datasets. For these two tasks, the specific PR and ROC curves of all methods on all datasets are given in Supplementary Figs S14–S26. From the experimental results, it can be seen that SFINN can effectively infer causal relationships between genes on both types of single-cell data.

### 3.5 Robustness of SFINN to sample size

Considering that the sample size of gene expression dataset may impact the performance of SFINN, we conducted down-sampling experiments to assess how SFINN performs in the interaction and causal relationship prediction tasks under varying numbers of cells. For all datasets, we randomly extracted cells to create subsets with different proportions, namely 20%, 40%, 60%, and 80%. Considering the top performers in the task of inferring gene interactions from single-cell transcriptomic data are CNNC, DNN, and MI, and the top model except SFINN on spatial transcriptomic data is GCNG, we compared SFINN with these methods using different sizes of datasets. Supplementary Figs S27 and S28 illustrate the AUPRC/AUROC medians of SFINN model and the compared methods for interaction prediction task on single-cell transcriptomic datasets and spatial transcriptomic datasets, respectively. Besides, considering that DREMI, DNN, and CNNC are suitable for gene causality inference on single-cell expression data and GCNG is applicable for inferring gene causality on spatial transcriptomic data, we compared SFINN with these methods using different sizes of datasets (Supplementary Figs S29 and S30). Observing the charts, it can be seen that there may be a slight decrease in performance as the cell number decreases, while overall SFINN tends to stabilize. When predicting gene interactions or causalities using a smaller sample size, even only 20% of cells from the original dataset, SFINN still exhibits a robust performance.

### 3.6 Prediction power of SFINN

To investigate the effectiveness of SFINN in predicting potential GRNs of unknown TFs, we applied SFINN on the hESC dataset. First, we trained SFINN using the training set of hESC previously used in the interaction task, and then used the trained models to infer potential TF–gene pairs not included in the training set. We randomly selected 18 TFs to predict. It is noted that these TF are completely different from the TFs in the training set. Among the top 100 interactions, ZNF143 and TBP genes are included most, therefore we focused on these two TFs. ZNF143 is the human homolog of the transcriptional activator of selenocysteine transfer RNA (tRNASec) gene, which mediates the incorporation of Sec into selenoproteins (Lu *et al.* 2012). As a TF, ZNF143 has also been shown to be associated with the survival, proliferation, differentiation, migration, and invasion of human glioma cells (Chen *et al.* 2023). TBP (TATA-binding protein) plays a critical role in the formation of transcription initiation complex, and its aberrant expression may induce neurological disorders (Ivanova *et al.* 2022). The top 10 interacting genes associated with ZNF143 and TBP are given in Supplementary Fig. S31A. We found that nine of the predicted top 10 interacting pairs of ZNF143 have been confirmed by the Harmonizome database (Rouillard *et al.* 2016), and the top 10 associated with TBP have all been confirmed.

As to the task of causality prediction, we performed the similar analysis by using the trained model to predict unknown TF–gene regulation and kept focusing on the two genes. The top 10 regulations associated with ZNF143 and TBP are given in Supplementary Fig. 31B. We found that eight of the predicted top 10 target genes of ZNF143 have been supported by the Harmonizome database, and seven of the top 10 target genes of TBP have been validated.

In addition, we found that some gene pairs, ZNF143 and BAIAP2, ZN143 and MSANTD4, TBP and CKAP2L, and TBP and EXOC3, were not shown to be related in the whole ground-truth, but were predicted to be related by SFINN and were validated by the external database. This indicates that SFINN has the ability of predicting novel gene regulations.

### 3.7 Ablation experiments

#### 3.7.1 Effect of SFN

To verify the effectiveness of the introduced SFN strategy in constructing cell–cell adjacency network, we compared the model built based on SFN with those built based on other commonly used methods for constructing cell–cell network, including PCC, Euclidean distance, Manhattan distance, and cosine distance, for all datasets and for TF–gene interaction and causality prediction tasks. We calculated the AUPRC and AUROC median on each dataset for each strategy (Supplementary Fig. S32), it can be known that overall, the model built based on the SFN strategy has a better performance, especially in the task of predicting gene causality.

#### 3.7.2 Effect of integrative neural network

**Figure 5.**
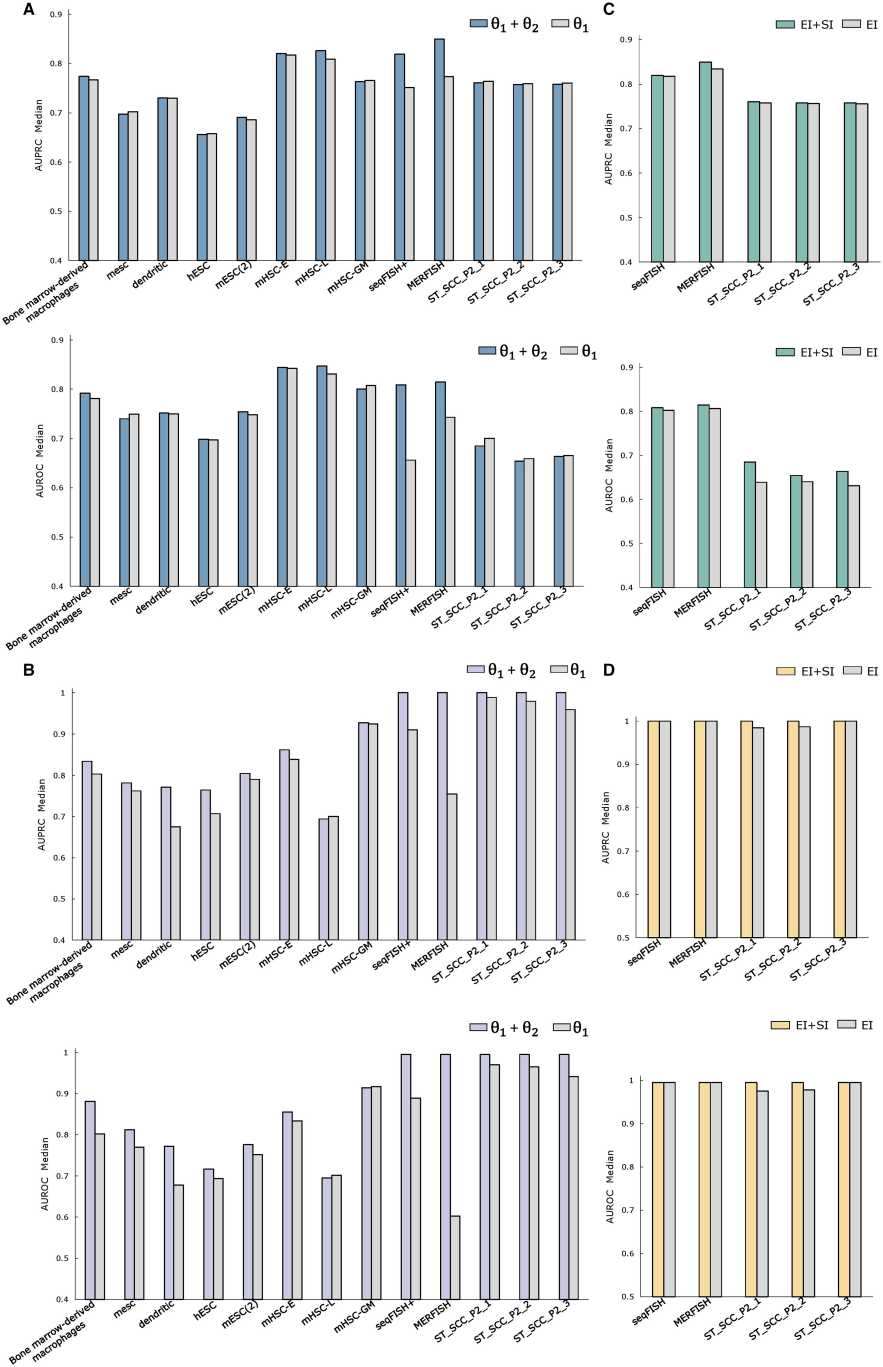
The results of ablation experiments of SFINN. The ablation experiment regarding the learned embedding *θ*2 from NN in the integrated neural network framework on all datasets for the tasks of (A) gene interaction and (B) causality prediction. The ablation experiment regarding the integration of spatial information (denoted as SI) and expression information (denoted as EI) on all spatial transcriptomic datasets for the tasks of (C) gene interaction and (D) causality prediction.

To investigate the effectiveness of designing an integrative neural network, i.e. introducing the ensemble neural network framework to combine the learned embeddings (*θ*1 and *θ*2) from GCN and NN, we conducted comparative experiments by removing NN on all datasets for TF–gene interaction and causal relationship prediction tasks. The experimental results for the two tasks are shown in Fig. 5A and B, respectively. The performance of using entire integrative neural network (i.e. combining *θ*1 and *θ*2) consistently outperforms the model involving only the GNN module (i.e. only using *θ*1) across multiple datasets, particularly noticeable on the seqFISH+ and MERFISH datasets and in the task of causality prediction. This suggests that integrating the NN module with the GCN module is beneficial for predicting interactions and regulations between genes.

#### 3.7.3 Effect of introducing spatial location

Furthermore, to validate the effectiveness of integrating expression and spatial information to construct a cell–cell adjacency matrix, we also conducted comparative experiments on all five spatial transcriptomic datasets for TF–gene interaction prediction (Fig. 5C) and causal relationship prediction tasks (Fig. 5D). In particular, on the three datasets within the ST_SCC_P2 series, the integration of spatial and expression information shows a more pronounced improvement in the AUROC median compared with using only the gene expression in the task of gene interaction prediction. The introduction of spatial information indeed facilitates the inference of GRNs to some extent.

## 4 Discussion

The inference of GRNs from single-cell transcriptomic data is a crucial research direction in current molecular biology and bioinformatics. The increase of single-cell and spatial transcriptomic data provides us with the opportunity to develop a supervised deep learning method for autonomously learning the feature representation of gene pair expression to better understand and interpret the relationships between genes. Considering that many of the existing GRN methods were not developed specifically for single-cell gene expression data, not being able to simultaneously opt for incorporating spatial location, and could not differentiate gene interactions and causalities, we introduced a supervised GRN inference framework named SFINN to predict potential interactions and regulations between genes from single-cell and spatial transcriptomic data. SFINN transforms the task of predicting gene regulations into an end-to-end binary classification problem.

In this study, SFINN was initially applied to eight scRNA-seq datasets and compared with various existing methods, demonstrating its superior performance in gene interaction task. It exhibited better results in terms of AUROC and AUPRC, showcasing accuracy and robustness in predicting gene interactions at the single-cell level. Notably, SFINN also exhibited outstanding performance when being applied to spatial transcriptomic datasets, surpassing some existing methods. Then, SFINN was used to the task of predicting gene causalities from both single-cell and spatial transcriptomic data. The results demonstrated SFINN has the ability of inferring directional gene relationships among genes. We also showcased the power of SFINN in predicting novel TF–gene interactions and causal relationships based on single-cell gene expression data.

The design of integrative network framework allows us to extract the feature representation of gene pair expression from multiple views, facilitating to better learn the relationships between genes. Utilizing SFN on gene expression and successfully integrating with spatial information, SFINN is able to construct a cellular neighborhood network accurately, therefore identifying gene regulations accurately. SFINN is an effective tool to infer gene regulations from single-cell and spatial transcriptomic data for uncovering gene regulatory mechanisms at the cellular level.

Despite the promising performance of SFINN, several issues can be addressed in the future. SFINN needs to construct cell–cell adjacency matrix. If the sample size of gene expression data is very large, SFINN may consume more memory and running time to perform graph convolution. This may be solved by subsampling or designing a better way to store and calculate on a large cell–cell adjacency matrix. Besides, for real data analysis, reducing the dependence of SFINN model on the training data and making it applicable for a new independent data will be our future work.

## Supplementary Material

btae433_Supplementary_Data

## Data Availability

SFINN can be accessed at GitHub: https://github.com/JGuan-lab/SFINN. Gene expression and ChIP-Seq data of bone marrow-derived macrophages, dendritic cells, and mesc were originally downloaded from: https://github.com/xiaoyeye/CNNC. Gene expression and ChIP-Seq data of hESC, mESC(2), mHSC-E, mHSC-GM, and mHSC-L can be downloaded at https://zenodo.org/record/4475471#.YBNvFZMzZTY. The seqFISH+ data and cell location file, as well as the true interacting ligand-receptor list, were downloaded from https://github.com/xiaoyeye/GCNG/. The MERFISH dataset and associated cell location file were downloaded from https://www.pnas.org/content/116/39/19490/tab-figuresdata. Gene expression information and cell location file for the three ST_SCC datasets were obtained from https://github.com/drieslab/spatial-datasets/tree/master/data/2020_ST_SCC. All analysed inputs and ground-truths are also deposited at: https://zenodo.org/records/10558871.
