# Supplementary material for: SFINN: inferring gene regulatory network from single-cell and spatial transcriptomic data with shared factor neighborhood and integrated neural network
Source: Bioinformatics. 2024 Jul 1;40(7):btae433. doi: 10.1093/bioinformatics/btae433 (PMC11236097; doi:10.1093/bioinformatics/btae433)
Supplement: btae433_Supplementary_Data [file btae433_supplementary_data.pdf]

Supplementary Materials

1. Single-cell transcriptomic datasets

We used eight single-cell transcriptomic datasets to assess the ability of SFINN in identifying transcription factor (TF)-target gene interactions and also causal relationships. Three single-cell transcriptomic datasets along with their corresponding ground-truth data were obtained from the study of Yuan et al (Yuan and Bar-Joseph, 2019), including datasets of bone marrow-derived macrophages (Alavi, et al., 2018), dendritic single cells (Alavi, et al., 2018) and IB10 mouse embryonic stem cells (Klein, et al., 2015). For the selection of TFs, we adhered to the previous practice (Yuan and Bar-Joseph, 2019). Additionally, we collected five single-cell transcriptomic datasets and their associated ground-truth data from the study conducted by Chen et al (Chen, et al., 2021), including datasets of 5G6GR mouse embryonic stem cells (Hayashi, et al., 2018), human embryonic stem cells (Chu, et al., 2016) and the three lineages of mouse hematopoietic stem cells (Nestorowa, et al., 2016). Regarding these datasets, we randomly selected 18 TFs from the overall ground-truth set like the way in the previous study (Chen, et al., 2021).

Bone marrow-derived macrophages (Alavi, et al., 2018). This dataset was obtained through single-cell transcriptome sequencing of mouse bone marrow cells. Bone marrow-derived macrophages are commonly used macrophages cultured in vitro and play a crucial role in immune regulation.

Dendritic single cells (Alavi, et al., 2018). Dendritic cells are a type of immune cell with the ability to recognize and present antigens, playing a vital role in the normal functioning of the immune system.

IB10 mouse embryonic stem cells (mesc) (Klein, et al., 2015). IB10 mouse embryonic stem cell is a widely used mouse embryonic stem cell line with self-renewal and pluripotent potential, capable of differentiating into various cell types such as cardiomyocytes, neurons, hepatocytes, etc.

5G6GR mouse embryonic stem cells (mESC (2)) (Hayashi, et al., 2018). This is a genetically edited series of mouse embryonic stem cells expressing a recombinant protein called 5G6GR due to specific gene modifications.

Human embryonic stem cells (hESC) (Chu, et al., 2016). This dataset involves human embryonic stem cells, typically comprising cell lines obtained from human embryos. It is used to study issues such as the differentiation and developmental processes of human embryonic stem cells, cell fate determination mechanisms, gene

regulatory networks, and more.

Mouse hematopoietic stem cells (mHSC) (Nestorowa, et al., 2016). This dataset includes hematopoietic stem cells and progenitor cells from mouse bone marrow. It encompasses data from three lineages: mHSC-E representing the erythroid lineage, mHSC-GM representing the granulocyte-monocyte lineage, and mHSC-L representing the lymphoid lineage. mHSC-E includes cell types like primitive erythrocytes and mature red blood cells; mHSC-GM includes neutrophils, monocytes, macrophages, etc.; mHSC-L includes T cells, B cells, and other lymphoid cell types. The datasets of three lineages were analyzed separately.

2. Single-cell spatial transcriptomic datasets

For the tasks of inferring gene interactions and causal relationships, we analyzed five single-cell spatial transcriptomic datasets, including seqFISH+ dataset (Eng, et al., 2019), MERFISH dataset (Xia, et al., 2019) and the datasets of three cryosections from a patient with squamous cell carcinoma (Ji, et al., 2020). We downloaded the list of real interacting ligand-receptor from Yuan et al (Yuan and Bar-Joseph, 2020). Normalization procedures for raw spatial gene expression data followed the steps of Eng et al and Yuan et al (Eng, et al., 2019; Yuan and Bar-Joseph, 2020).

seqFISH+ dataset (Eng, et al., 2019). This dataset originates from experiments on gene expression in mouse cortical tissue. It involves the analysis of 10,000 genes across 913 cells in the mouse cortex.

MERFISH dataset (Xia, et al., 2019). This dataset includes expression information for 10,050 genes across 1,368 cells.

Datasets of three cryosections from a patient with squamous cell carcinoma (Ji, et al., 2020). These datasets correspond to 666, 646, and 638 cells, with 17,138, 17,344, and 17,833 genes expressed, respectively. We denoted them as ST_SCC_P2_1, ST_SCC_P2_2, and ST_SCC_P2_3. These datasets were analyzed separately.

References

Alavi, A.*, et al.* A web server for comparative analysis of single-cell RNA-seq data. *Nature Communications* 2018;9(1):4768.

Chen, J.*, et al.* DeepDRIM: a deep neural network to reconstruct cell-type-specific gene regulatory network using single-cell RNA-seq data. *Briefings in Bioinformatics* 2021;22(6):bbab325.

Chu, L.-F.*, et al.* Single-cell RNA-seq reveals novel regulators of human embryonic stem cell differentiation to definitive endoderm. *Genome Biology* 2016;17(1):173.

Eng, C.-H.L., *et al.* Transcriptome-scale super-resolved imaging in tissues by RNA seqFISH+. *Nature* 2019;568(7751):235-239.

Hayashi, T., *et al.* Single-cell full-length total RNA sequencing uncovers dynamics of recursive splicing and enhancer RNAs. *Nature Communications* 2018;9(1):619.

Ji, A.L., *et al.* Multimodal Analysis of Composition and Spatial Architecture in Human Squamous Cell Carcinoma. *Cell* 2020;182(2):497-514.e422.

Klein, Allon M., *et al.* Droplet Barcoding for Single-Cell Transcriptomics Applied to Embryonic Stem Cells. *Cell* 2015;161(5):1187-1201.

Nestorowa, S., *et al.* A single-cell resolution map of mouse hematopoietic stem and progenitor cell differentiation. *Blood* 2016;128(8):e20-e31.

Xia, C., *et al.* Spatial transcriptome profiling by MERFISH reveals subcellular RNA compartmentalization and cell cycle-dependent gene expression. *Proceedings of the National Academy of Sciences* 2019;116(39):19490-19499.

Yuan, Y. and Bar-Joseph, Z. Deep learning for inferring gene relationships from single-cell expression data. *Proc Natl Acad Sci U S A* 2019;116(52):27151-27158.

Yuan, Y. and Bar-Joseph, Z. GCNG: graph convolutional networks for inferring gene interaction from spatial transcriptomics data. *Genome Biology* 2020;21(1):300.

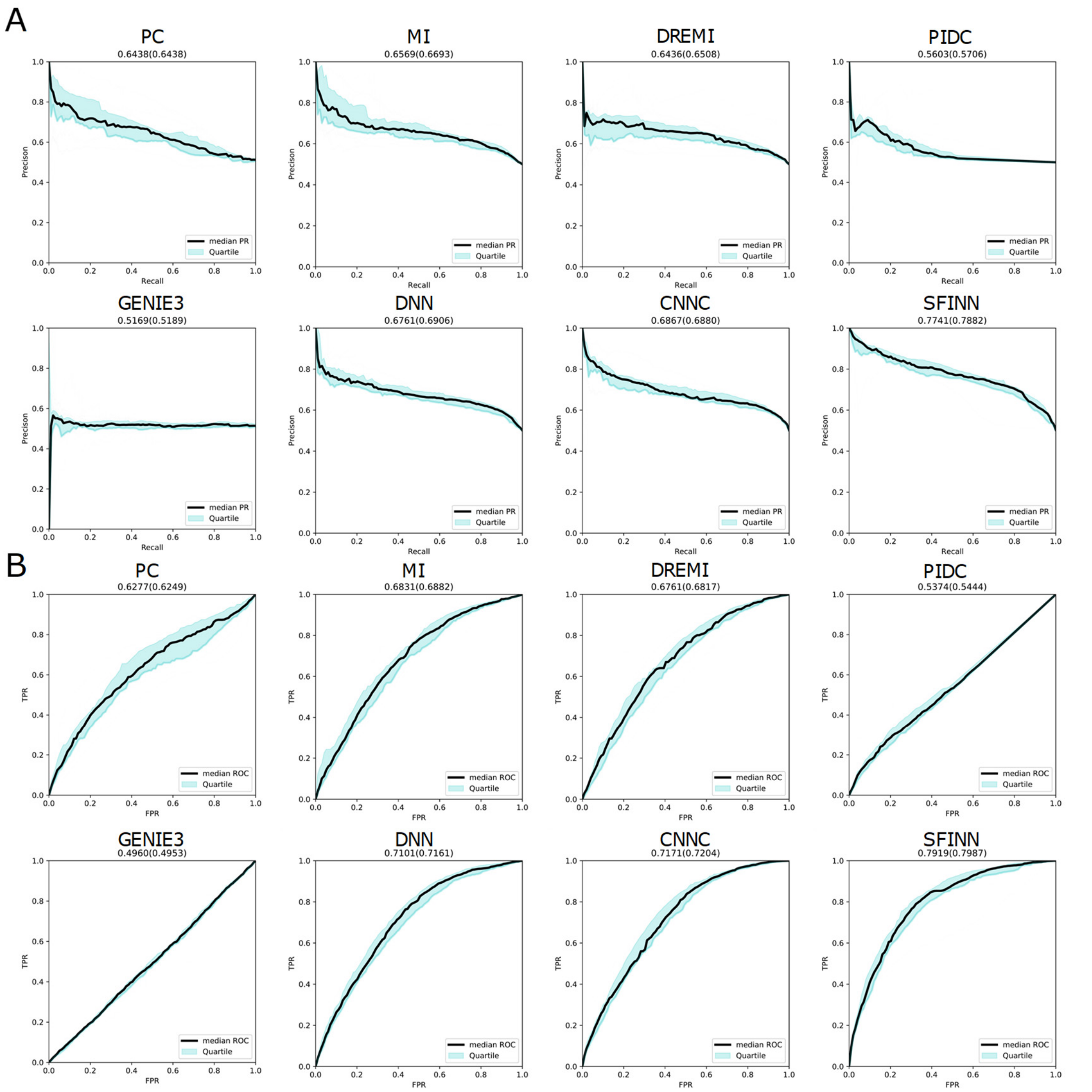

Supplementary Figure 1. (A) PR and (B) ROC curves of PC, MI, DREMI, PIDC, GENIE3, DNN, CNNC, and SFINN in predicting transcription factor-target gene interactions on bone marrow-derived macrophages dataset. The AUPRC/AUROC median (mean) across gene pairs of all transcription factors are shown above each graph.

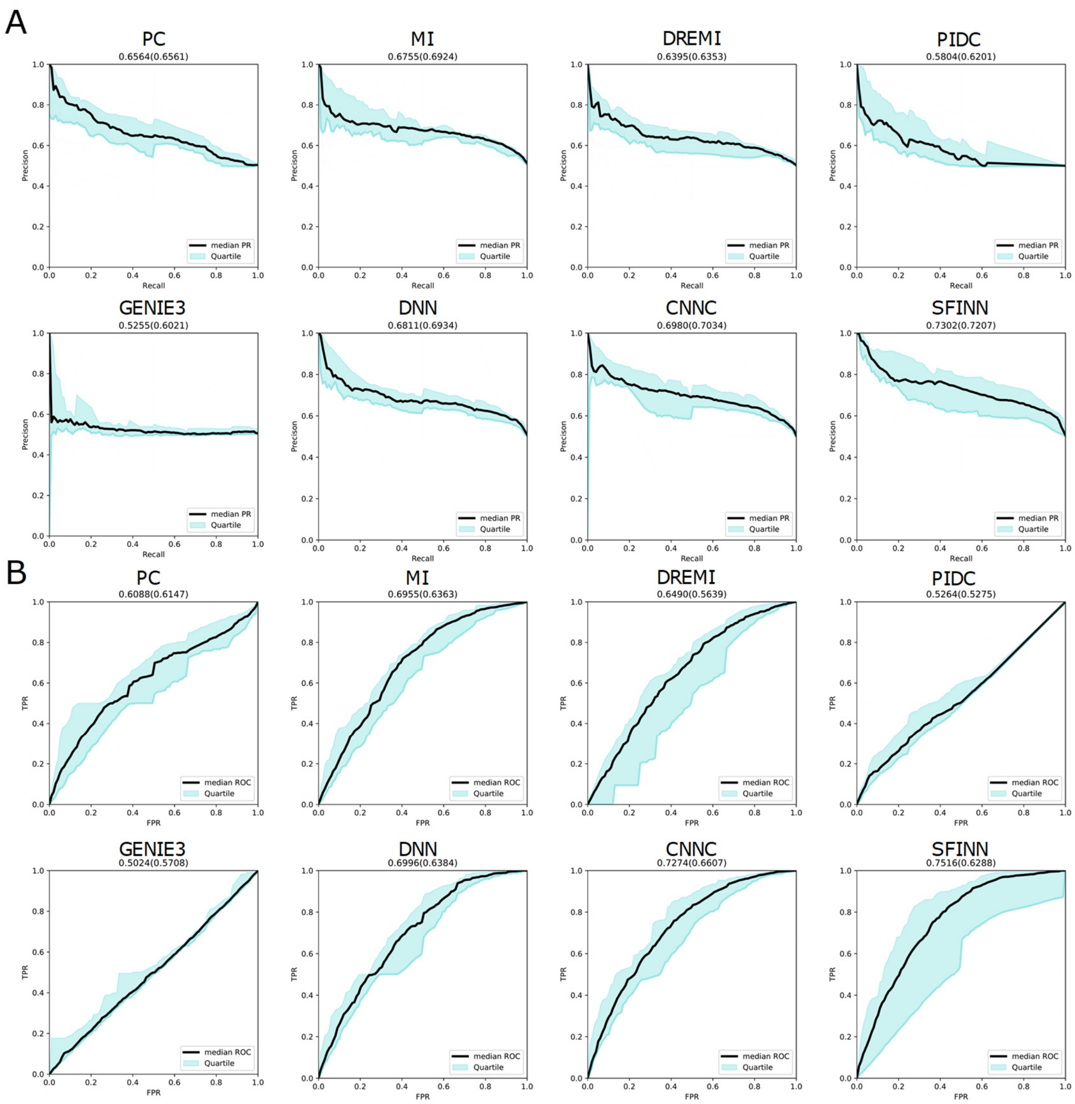

Supplementary Figure 2. (A) PR and (B) ROC curves of PC, MI, DREMI, PIDC, GENIE3, DNN, CNNC, and SFINN in predicting transcription factor-target gene interactions on dendritic dataset. The AUPRC/AUROC median (mean) across gene pairs of all transcription factors are shown above each graph.

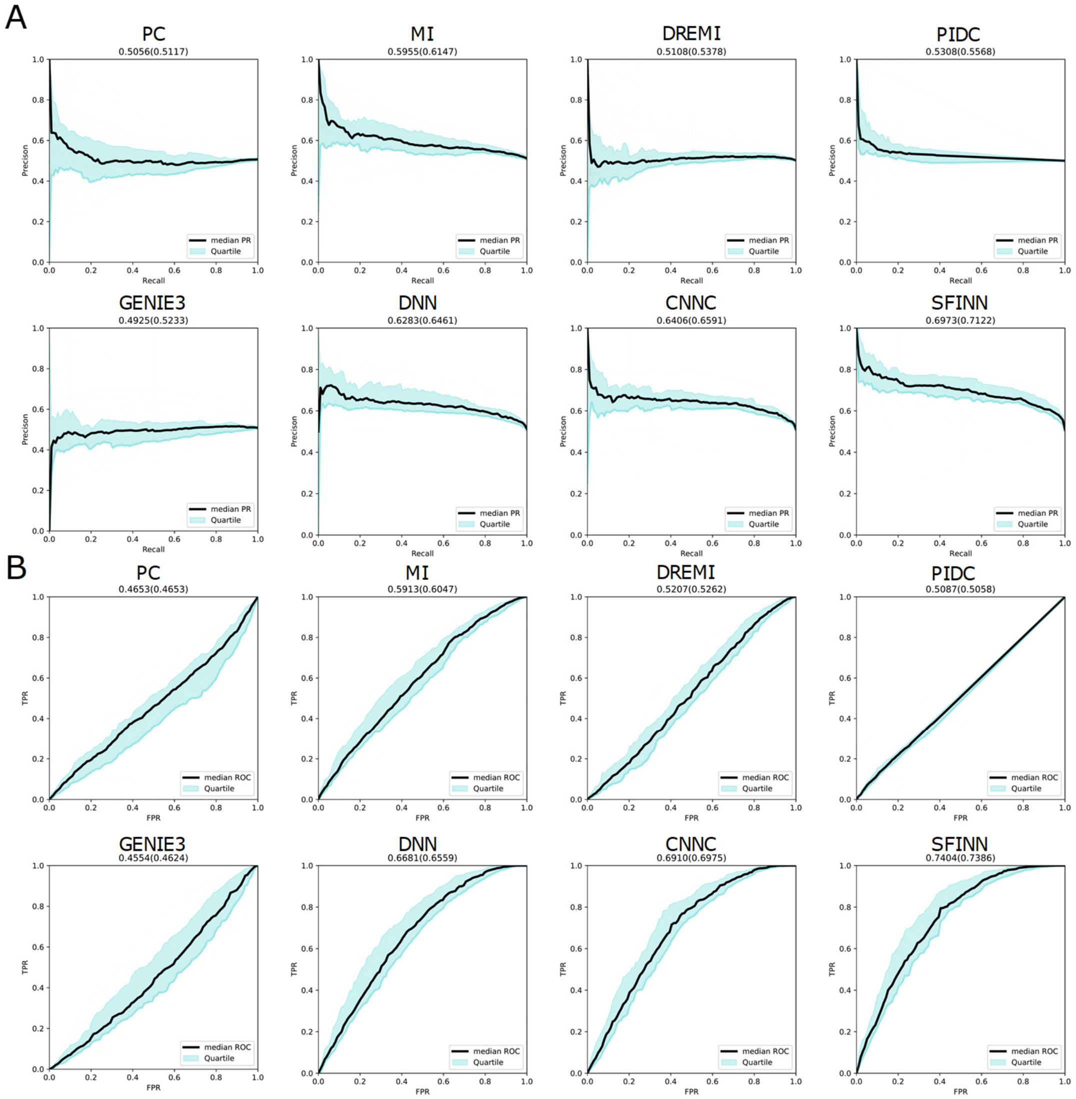

Supplementary Figure 3. (A) PR and (B) ROC curves of PC, MI, DREMI, PIDC, GENIE3, DNN, CNNC, and SFINN in predicting transcription factor-target gene interactions on mesc dataset. The AUPRC/AUROC median (mean) across gene pairs of all transcription factors are shown above each graph.

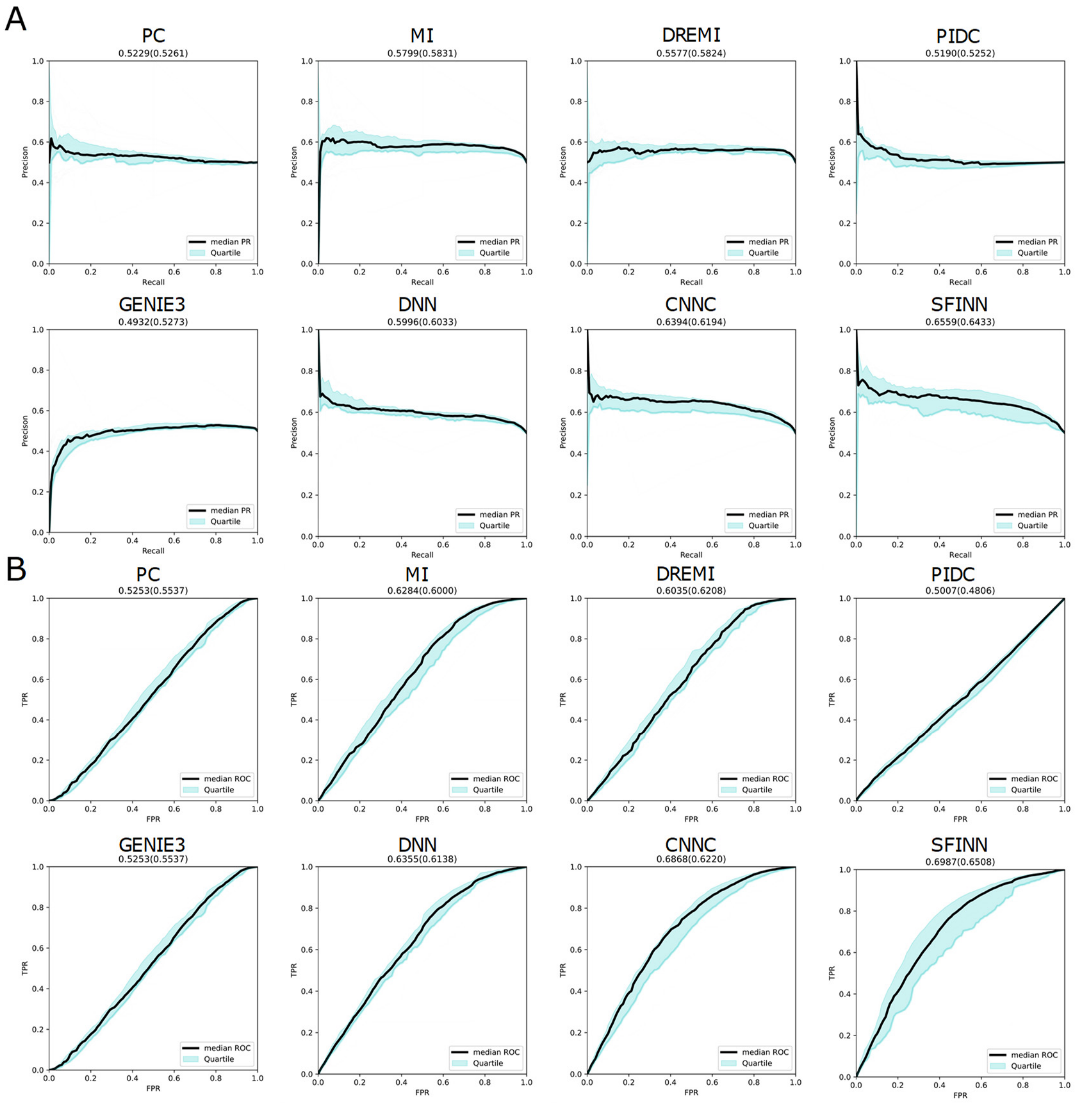

Supplementary Figure 4. (A) PR and (B) ROC curves of PC, MI, DREMI, PIDC, GENIE3, DNN, CNNC, and SFINN in predicting transcription factor-target gene interactions on hESC dataset. The AUPRC/AUROC median (mean) across gene pairs of all transcription factors are shown above each graph.

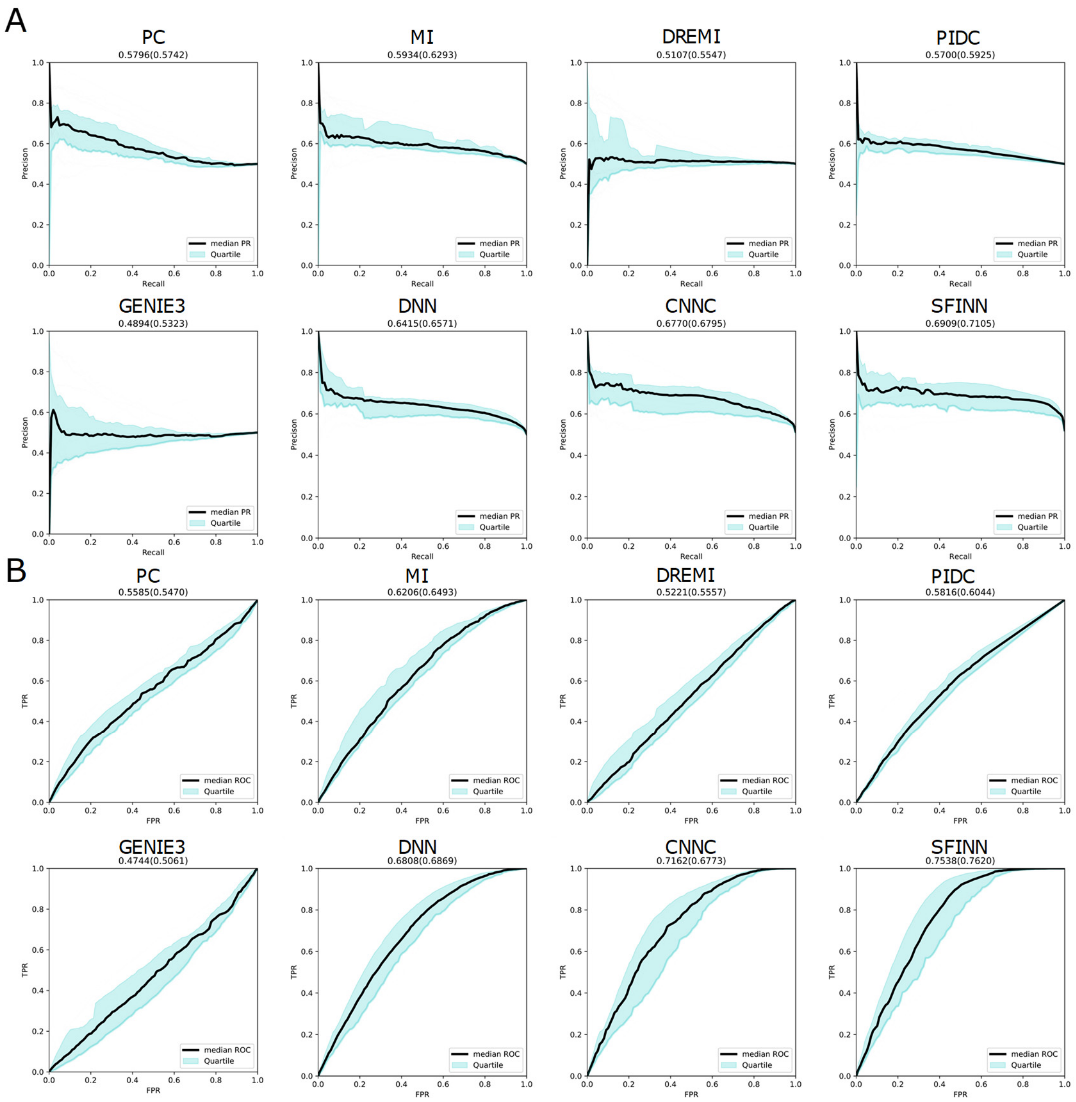

Supplementary Figure 5. (A) PR and (B) ROC curves of PC, MI, DREMI, PIDC, GENIE3, DNN, CNNC, and SFINN in predicting transcription factor-target gene interactions on mESC(2) dataset. The AUPRC/AUROC median (mean) across gene pairs of all transcription factors are shown above each graph.

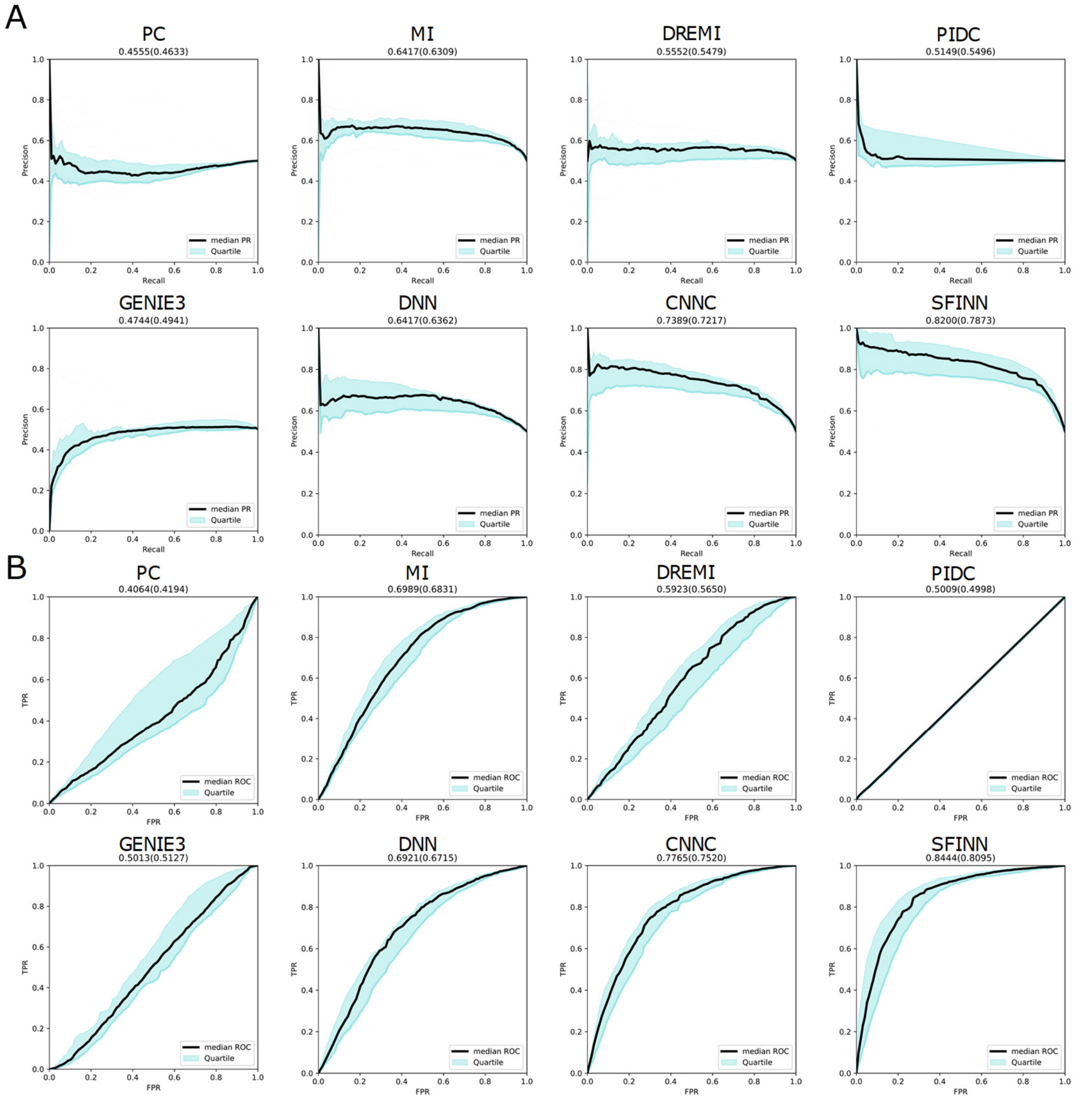

Supplementary Figure 6. (A) PR and (B) ROC curves of PC, MI, DREMI, PIDC, GENIE3, DNN, CNNC, and SFINN in predicting transcription factor-target gene interactions on mHSC-E dataset. The AUPRC/AUROC median (mean) across gene pairs of all transcription factors are shown above each graph.

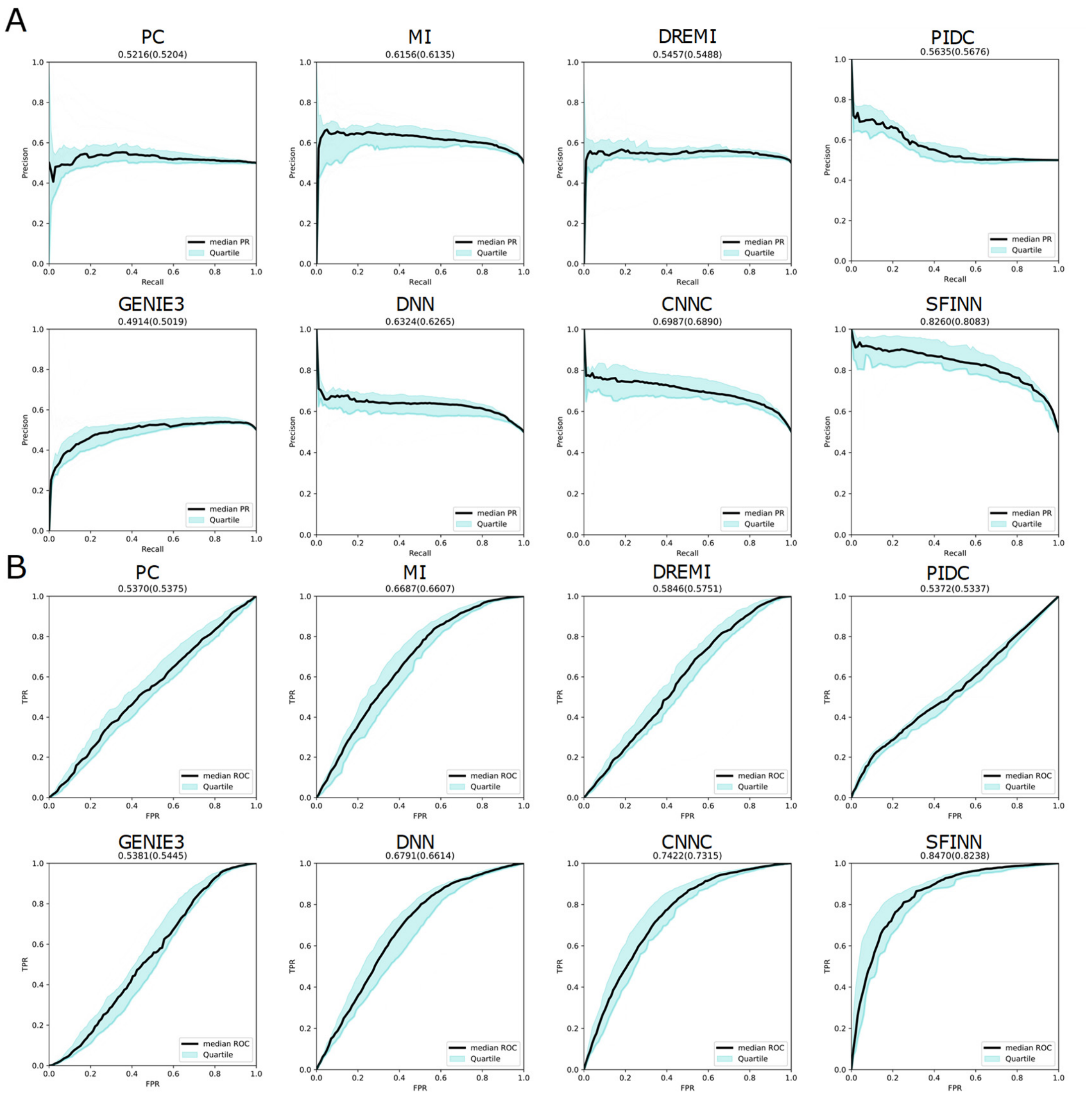

Supplementary Figure 7. (A) PR and (B) ROC curves of PC, MI, DREMI, PIDC, GENIE3, DNN, CNNC, and SFINN in predicting transcription factor-target gene interactions on mHSC-L dataset. The AUPRC/AUROC median (mean) across gene pairs of all transcription factors are shown above each graph.

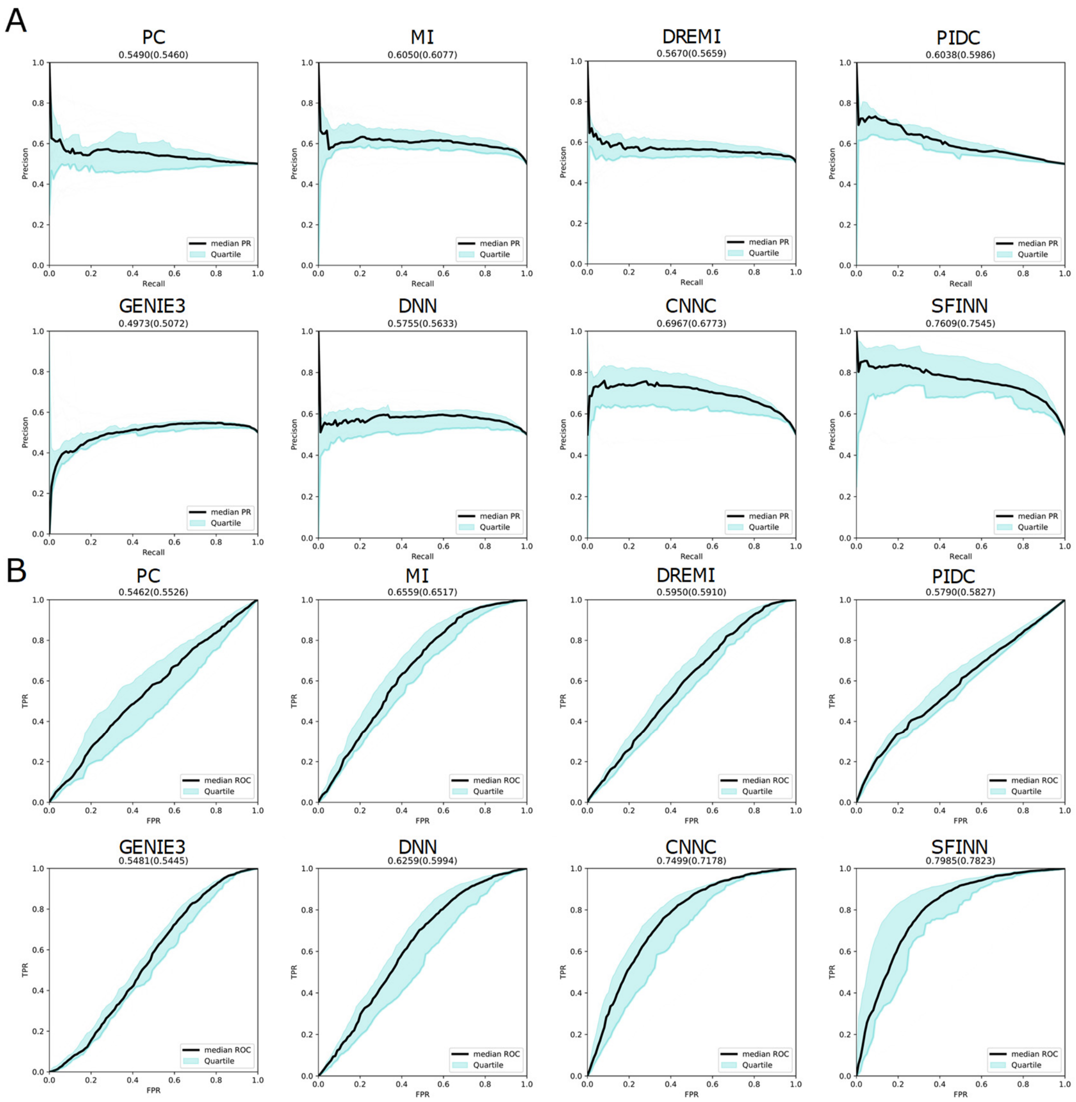

Supplementary Figure 8. (A) PR and (B) ROC curves of PC, MI, DREMI, PIDC, GENIE3, DNN, CNNC, and SFINN in predicting transcription factor-target gene interactions on mHSC-GM dataset. The AUPRC/AUROC median (mean) across gene pairs of all transcription factors are shown above each graph.

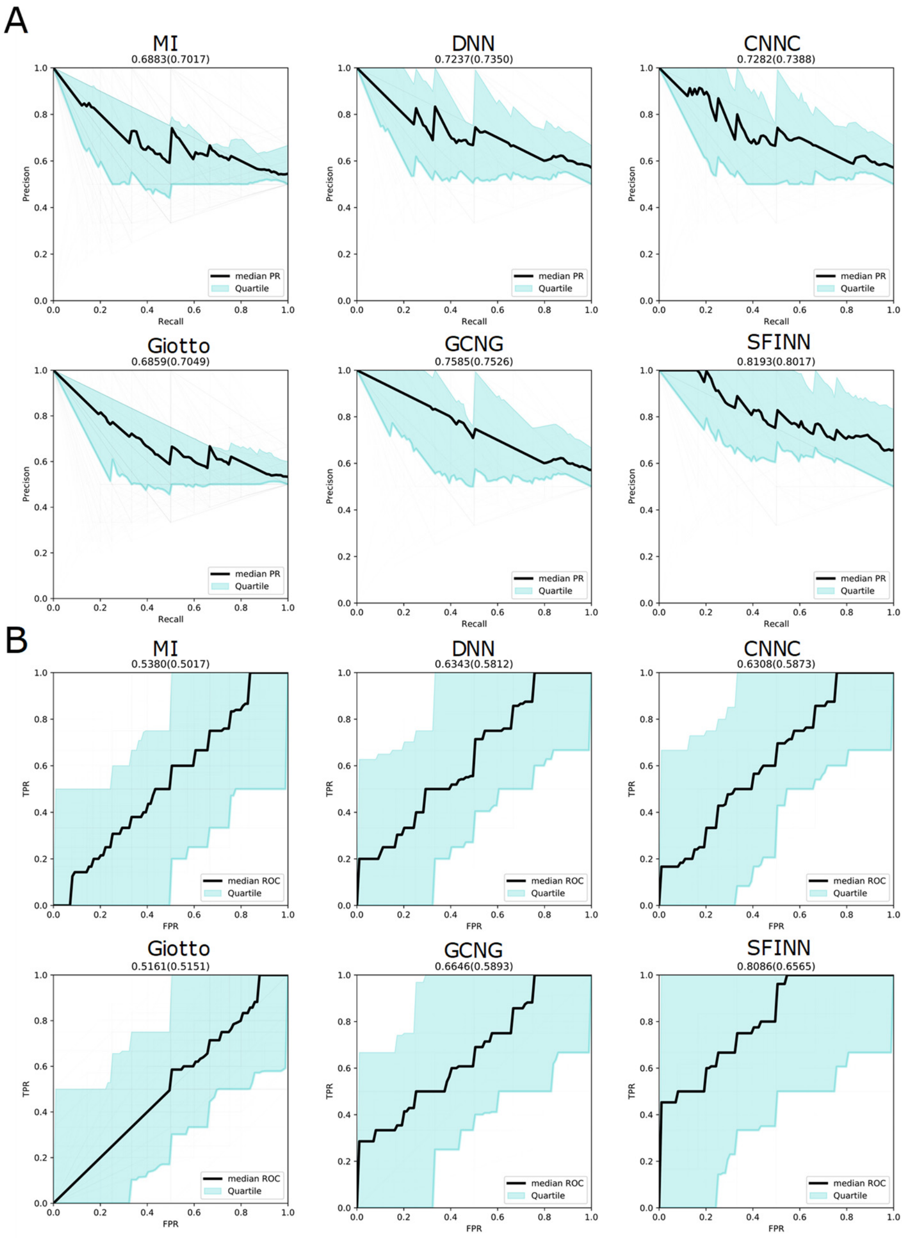

Supplementary Figure 9. (A) PR and (B) ROC curves of MI, DNN, CNNC, Giotto, GCNG, and SFINN in predicting transcription factor-target gene interactions on seqFISH+ dataset. The AUPRC/AUROC median (mean) across gene pairs of all transcription factors are shown above each graph.

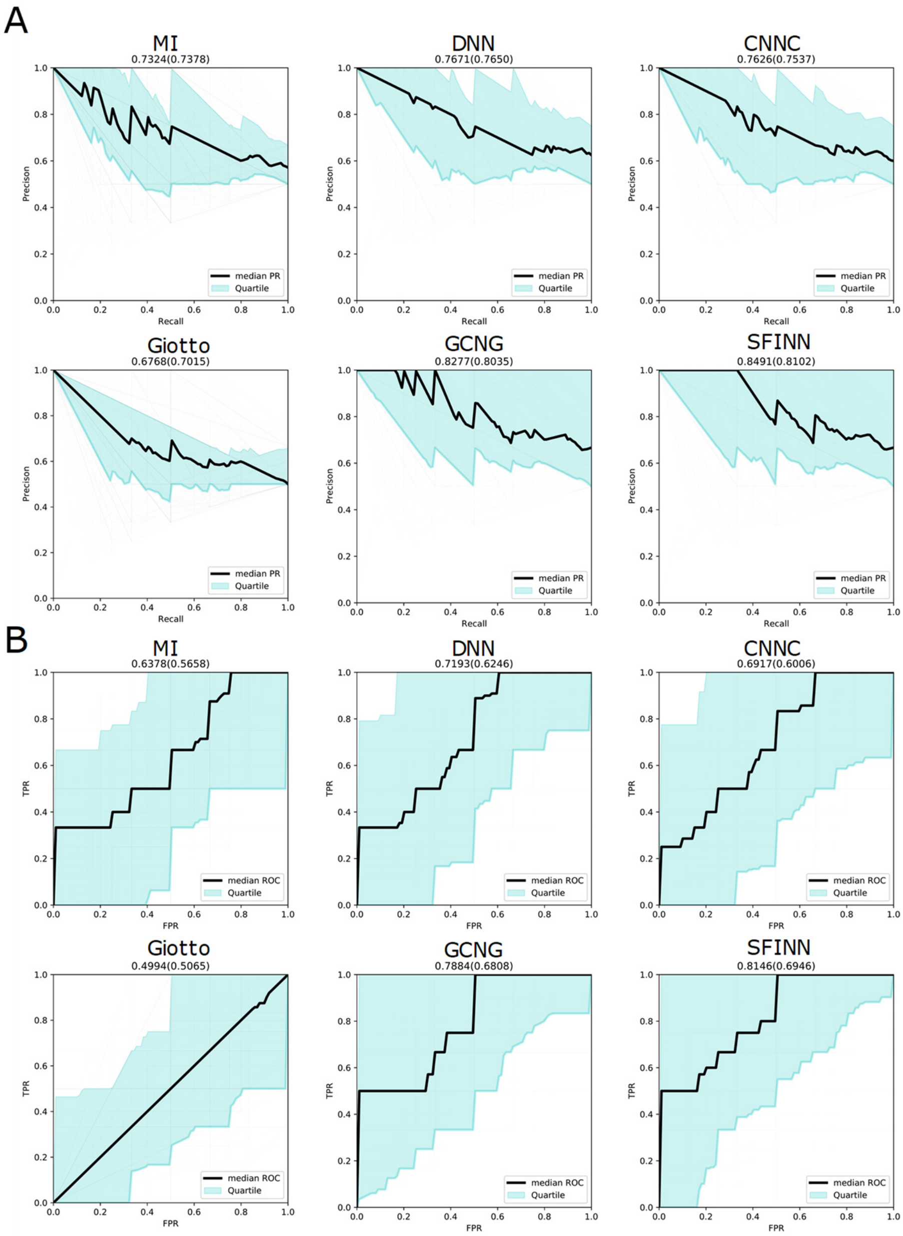

Supplementary Figure 10. (A) PR and (B) ROC curves of MI, DNN, CNNC, Giotto, GCNG, and SFINN in predicting transcription factor-target gene interactions on MERFISH dataset. The AUPRC/AUROC median (mean) across gene pairs of all transcription factors are shown above each graph.

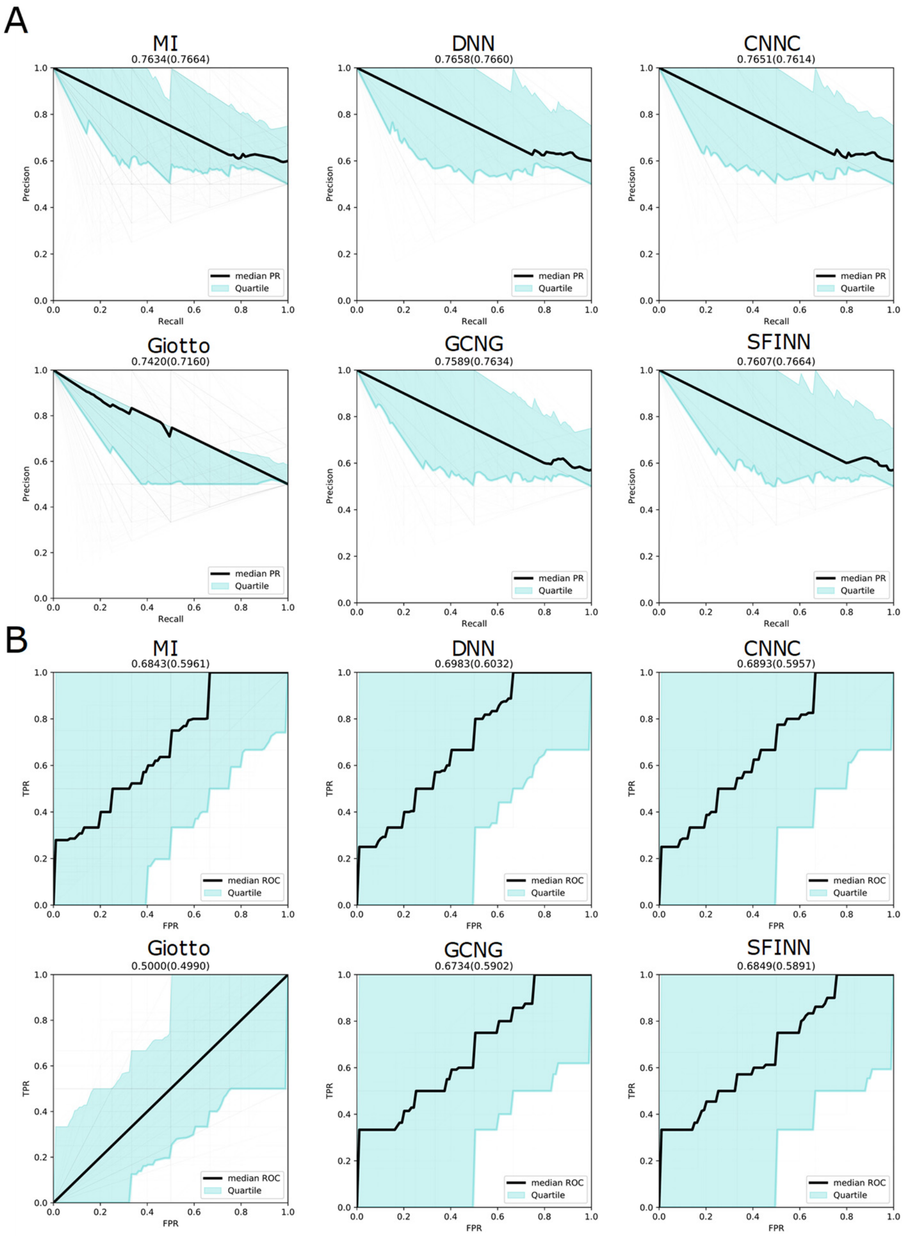

Supplementary Figure 11. (A) PR and (B) ROC curves of MI, DNN, CNNC, Giotto, GCNG, and SFINN in predicting transcription factor-target gene interactions on ST_SCC_P2_1 dataset. The AUPRC/AUROC median (mean) across gene pairs of all transcription factors are shown above each graph.

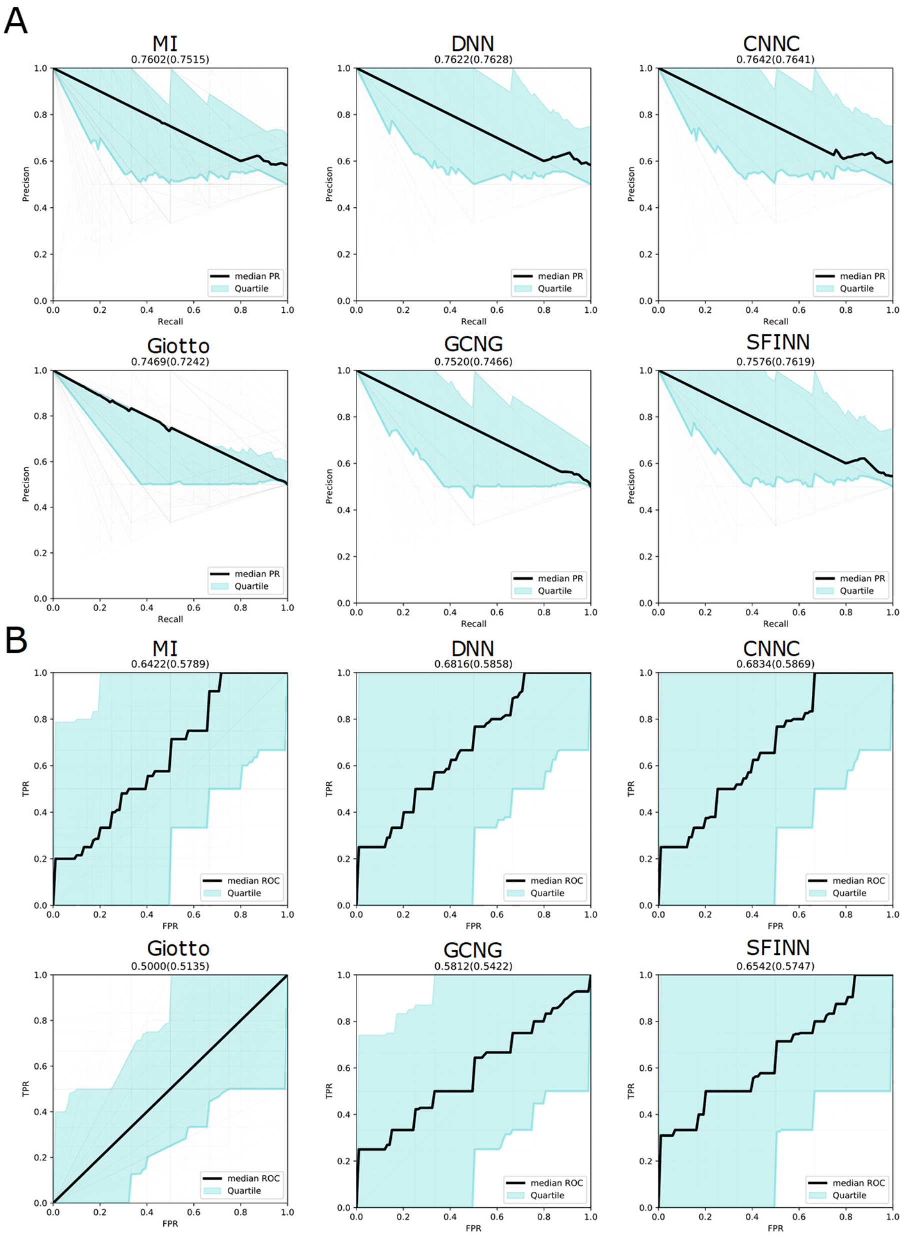

Supplementary Figure 12. (A) PR and (B) ROC curves of MI, DNN, CNNC, Giotto, GCNG, and SFINN in predicting transcription factor-target gene interactions on ST_SCC_P2_2 dataset. The AUPRC/AUROC median (mean) across gene pairs of all transcription factors are shown above each graph.

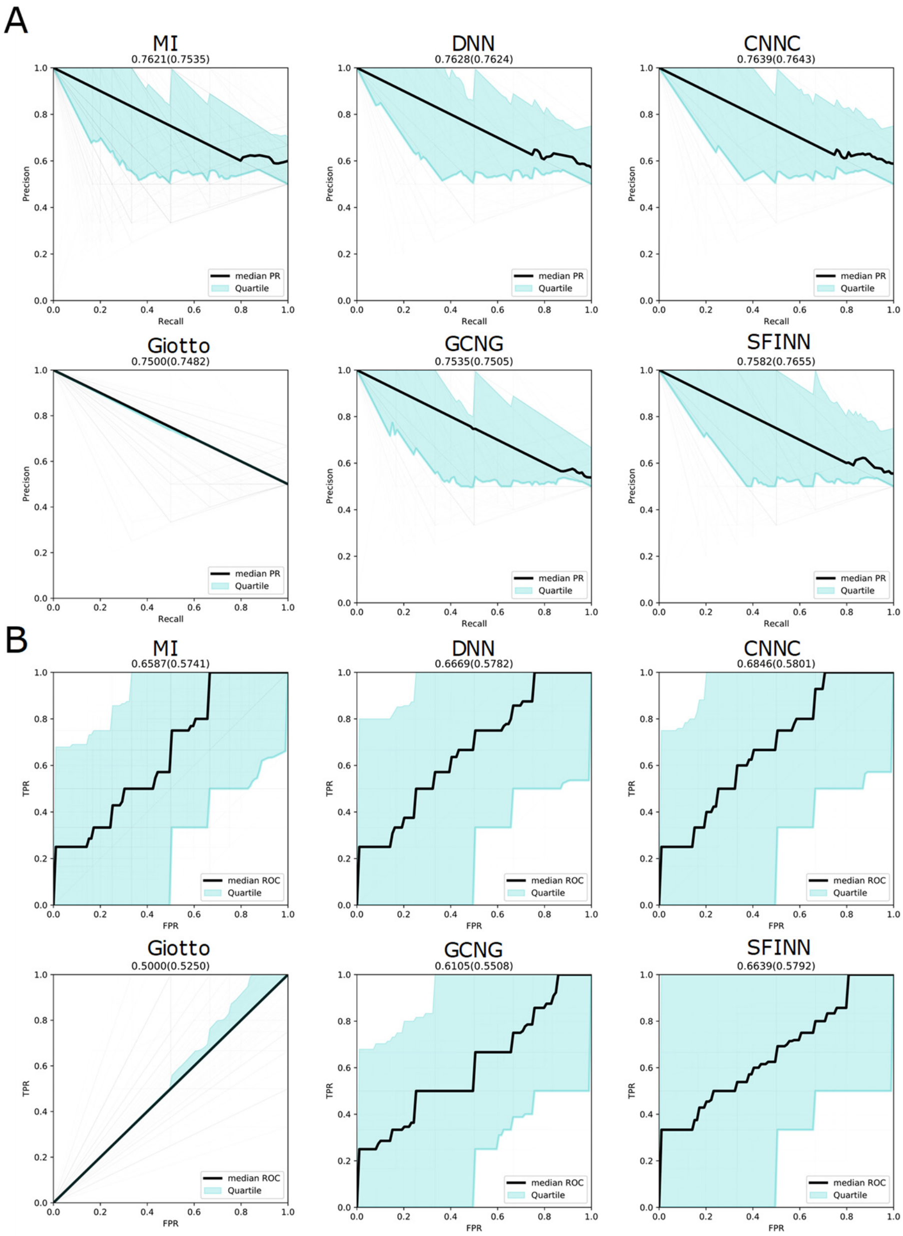

Supplementary Figure 13. (A) PR and (B) ROC curves of MI, DNN, CNNC, Giotto, GCNG, and SFINN in predicting transcription factor-target gene interactions on ST_SCC_P2_3 dataset. The AUPRC/AUROC median (mean) across gene pairs of all transcription factors are shown above each graph.

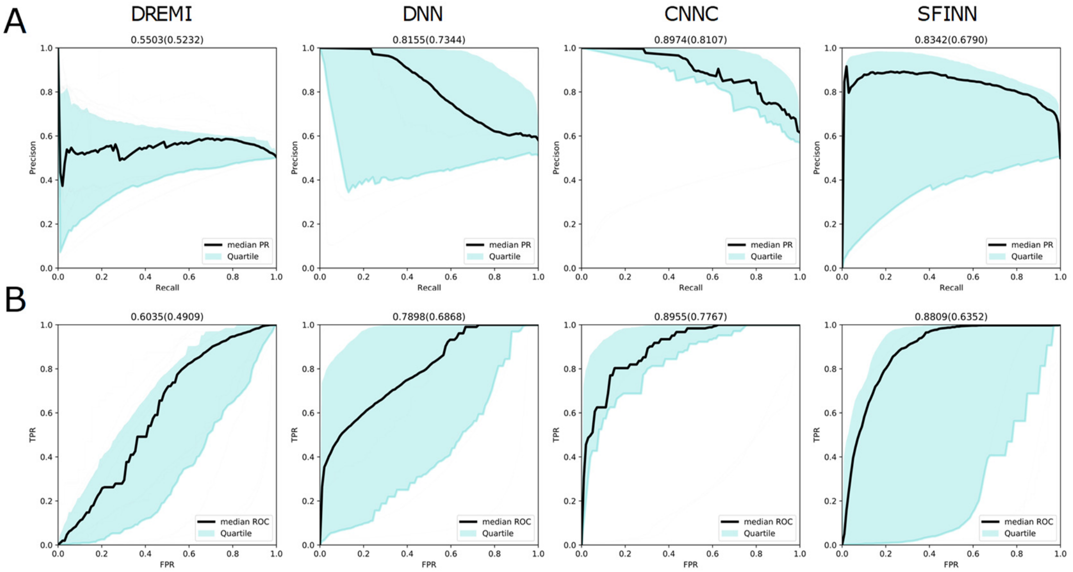

Supplementary Figure 14. (A) PR and (B) ROC curves of DREMI, DNN, CNNC, and SFINN in predicting transcription factor-target gene causalities on bone marrow-derived macrophage dataset. The AUPRC/AUROC median (mean) across gene pairs of all transcription factors are shown above each graph.

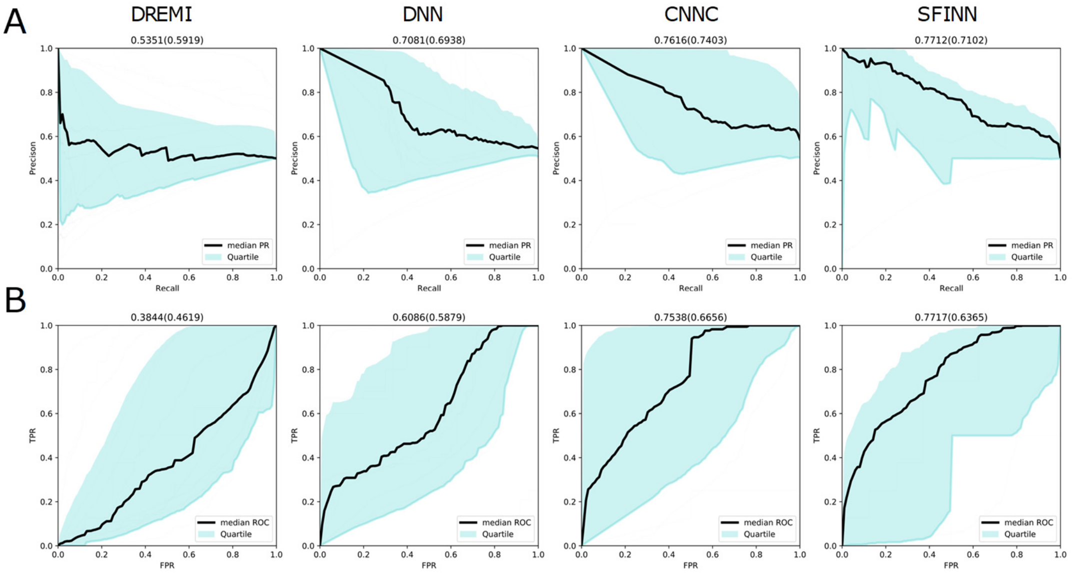

Supplementary Figure 15. (A) PR and (B) ROC curves of DREMI, DNN, CNNC, and SFINN in predicting transcription factor-target gene causalities on dendritic dataset. The AUPRC/AUROC median (mean) across gene pairs of all transcription factors are shown above each graph.

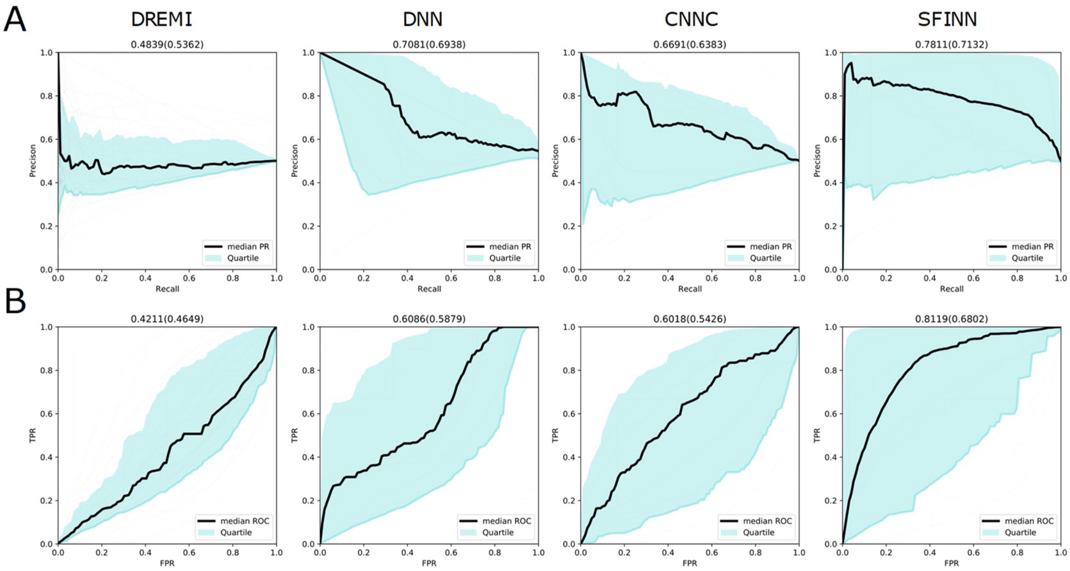

Supplementary Figure 16. (A) PR and (B) ROC curves of DREMI, DNN, CNNC, and SFINN in predicting transcription factor-target gene causalities on mesc dataset. The AUPRC/AUROC median (mean) across gene pairs of all transcription factors are shown above each graph.

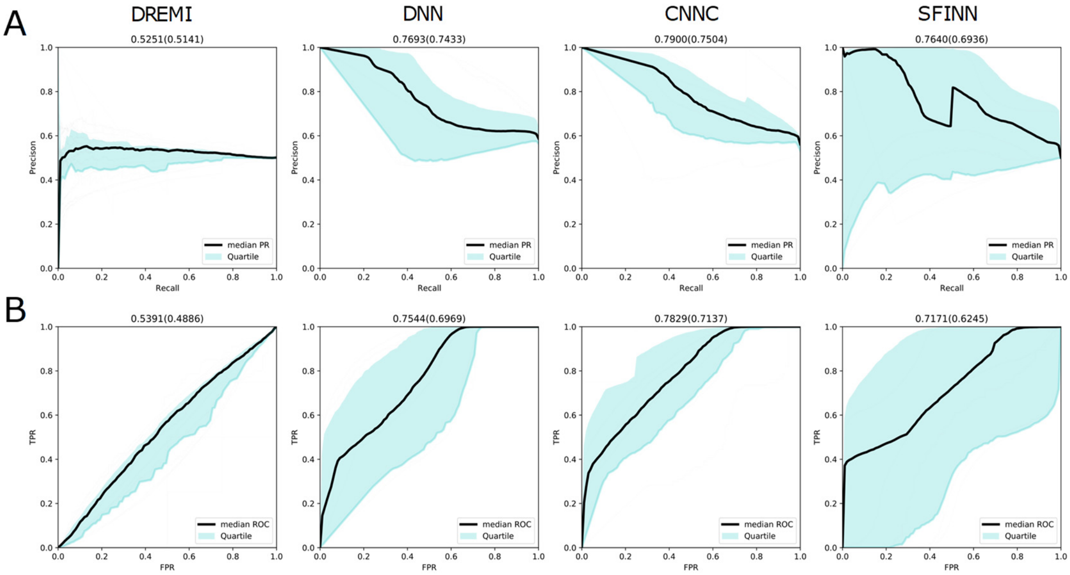

Supplementary Figure 17. (A) PR and (B) ROC curves of DREMI, DNN, CNNC, and SFINN in predicting transcription factor-target gene causalities on hESC dataset. The AUPRC/AUROC median (mean) across gene pairs of all transcription factors are shown above each graph.

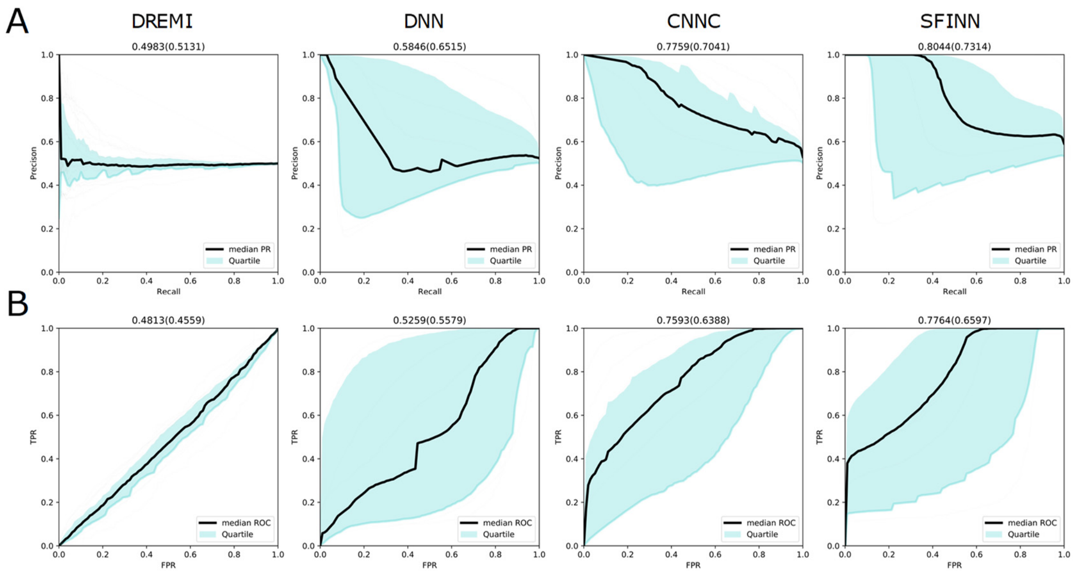

Supplementary Figure 18. (A) PR and (B) ROC curves of DREMI, DNN, CNNC, and SFINN in predicting transcription factor-target gene causalities on mESC(2) dataset. The AUPRC/AUROC median (mean) across gene pairs of all transcription factors are shown above each graph.

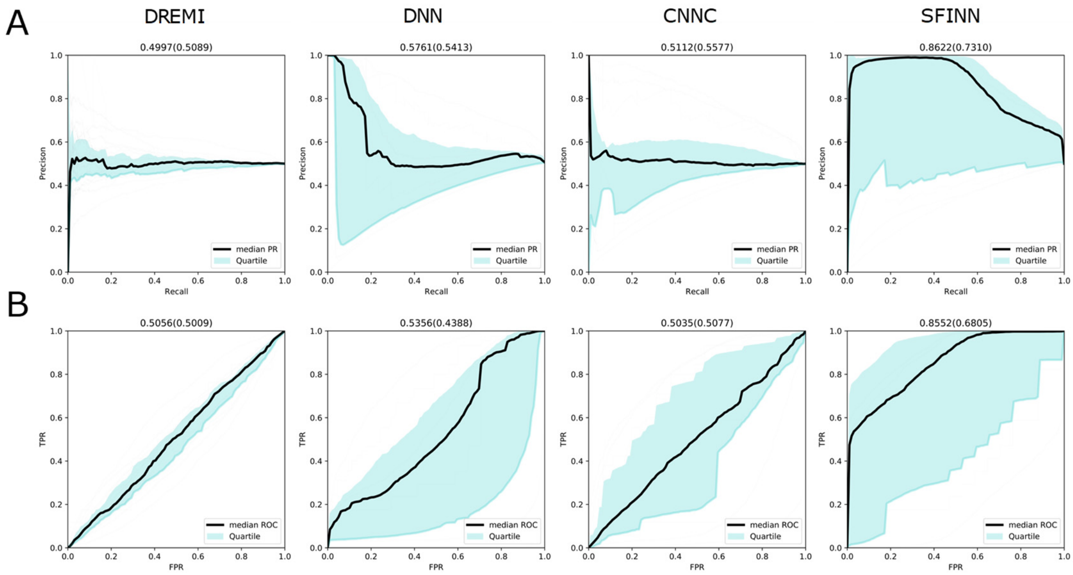

Supplementary Figure 19. (A) PR and (B) ROC curves of DREMI, DNN, CNNC, and SFINN in predicting transcription factor-target gene causalities on mHSC-E dataset. The AUPRC/AUROC median (mean) across gene pairs of all transcription factors are shown above each graph.

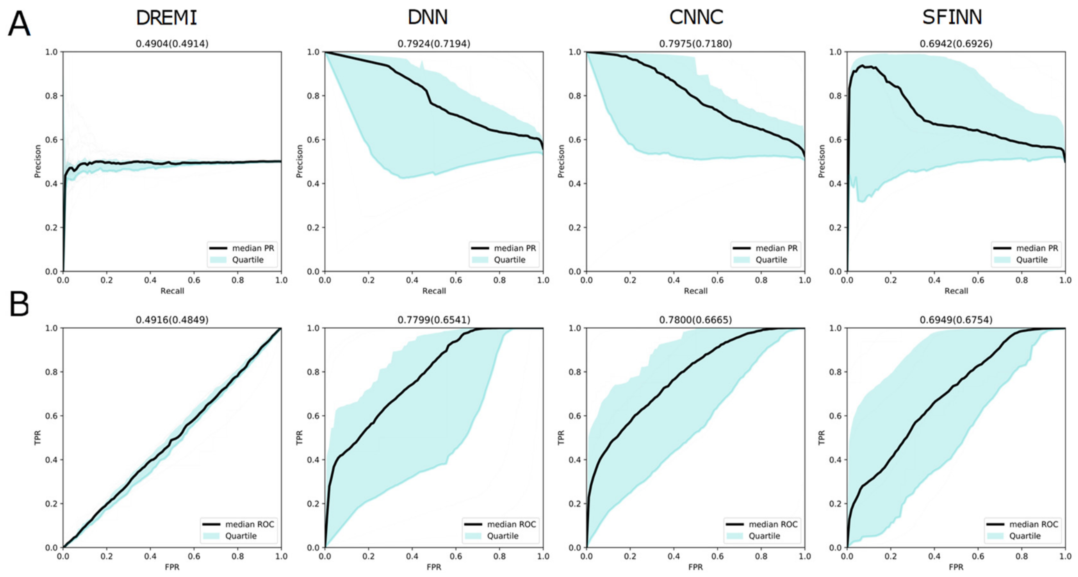

Supplementary Figure 20. (A) PR and (B) ROC curves of DREMI, DNN, CNNC, and SFINN in predicting transcription factor-target gene causalities on mHSC-L dataset. The AUPRC/AUROC median (mean) across gene pairs of all transcription factors are shown above each graph.

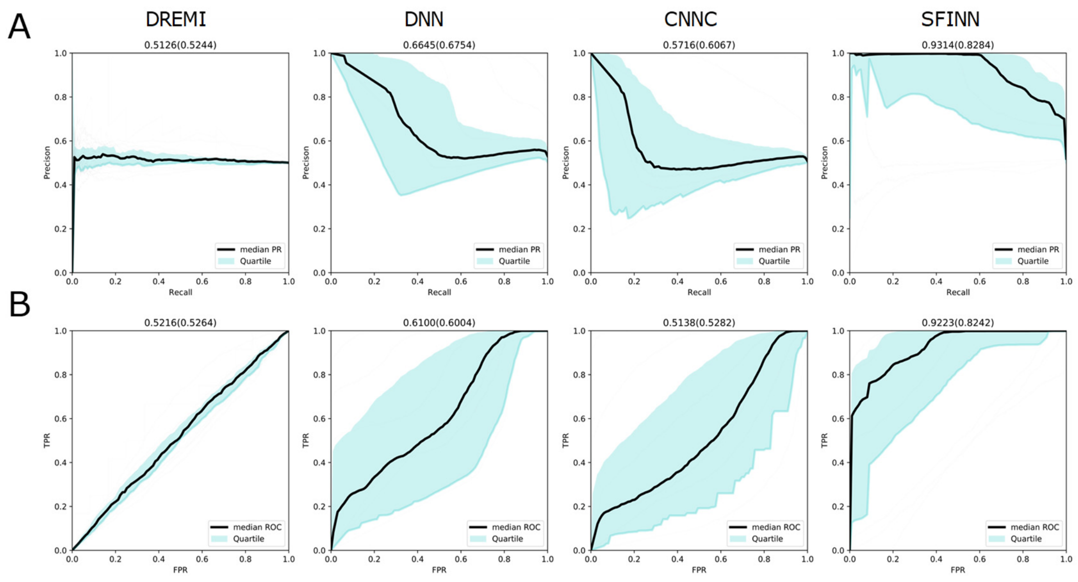

Supplementary Figure 21. (A) PR and (B) ROC curves of DREMI, DNN, CNNC, and SFINN in predicting transcription factor-target gene causalities on mHSC-GM dataset. The AUPRC/AUROC median (mean) across gene pairs of all transcription factors are shown above each graph.

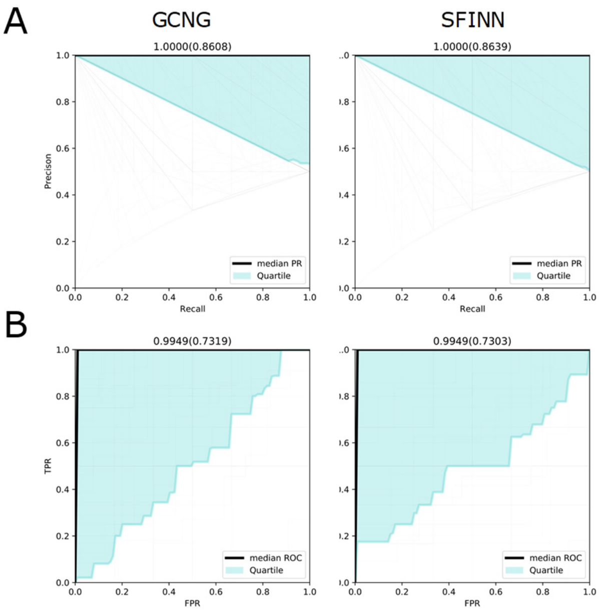

Supplementary Figure 22. (A) PR and (B) ROC curves of GCNG and SFINN in predicting transcription factor-target gene causalities on seqFISH+ dataset. The AUPRC/AUROC median (mean) across gene pairs of all transcription factors are shown above each graph.

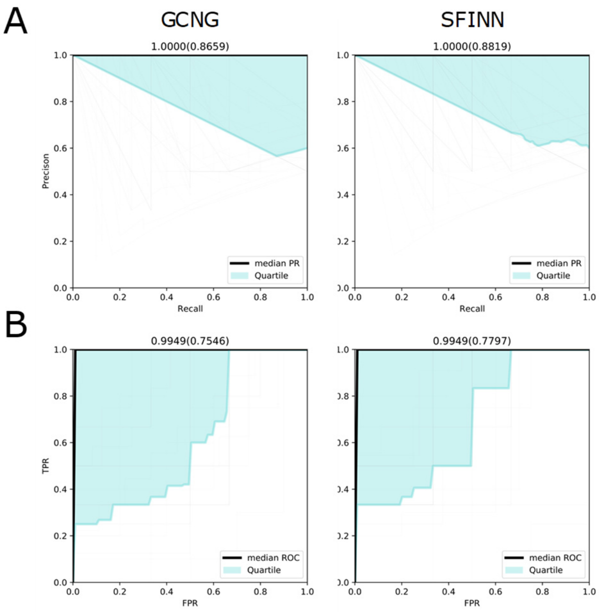

Supplementary Figure 23. (A) PR and (B) ROC curves of GCNG and SFINN in predicting transcription factor-target gene causalities on MERFISH dataset. The AUPRC/AUROC median (mean) across gene pairs of all transcription factors are shown above each graph.

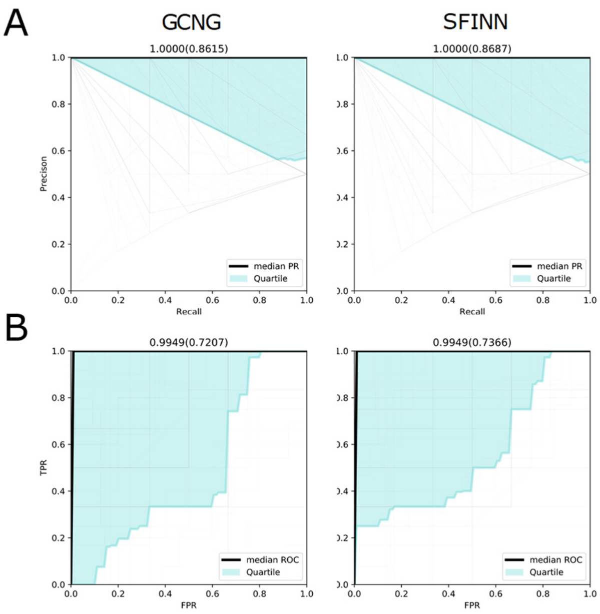

Supplementary Figure 24. (A) PR and (B) ROC curves of GCNG and SFINN in predicting transcription factor-target gene causalities on ST_SCC_P2_1 dataset. The AUPRC/AUROC median (mean) across gene pairs of all transcription factors are shown above each graph.

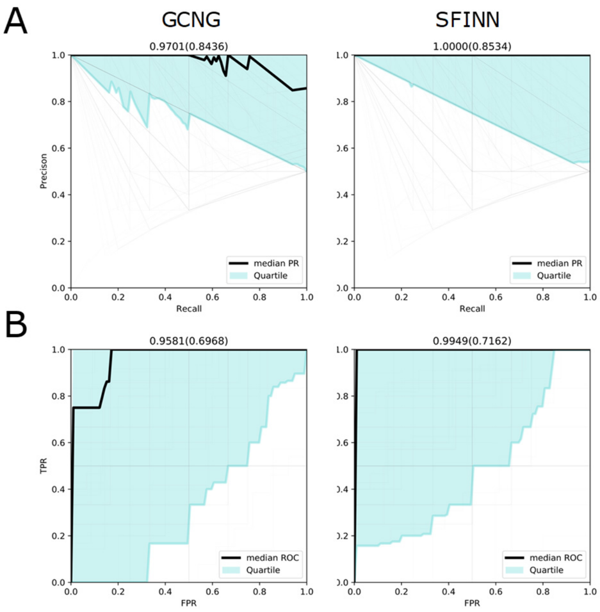

Supplementary Figure 25. (A) PR and (B) ROC curves of GCNG and SFINN in predicting transcription factor-target gene causalities on ST_SCC_P2_2 dataset. The AUPRC/AUROC median (mean) across gene pairs of all transcription factors are shown above each graph.

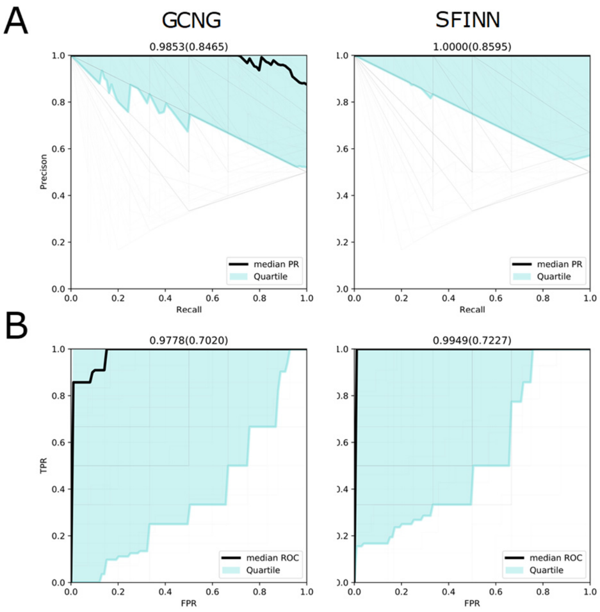

Supplementary Figure 26. (A) PR and (B) ROC curves of GCNG and SFINN in predicting transcription factor-target gene causalities on ST_SCC_P2_3 dataset. The AUPRC/AUROC median (mean) across gene pairs of all transcription factors are shown above each graph.

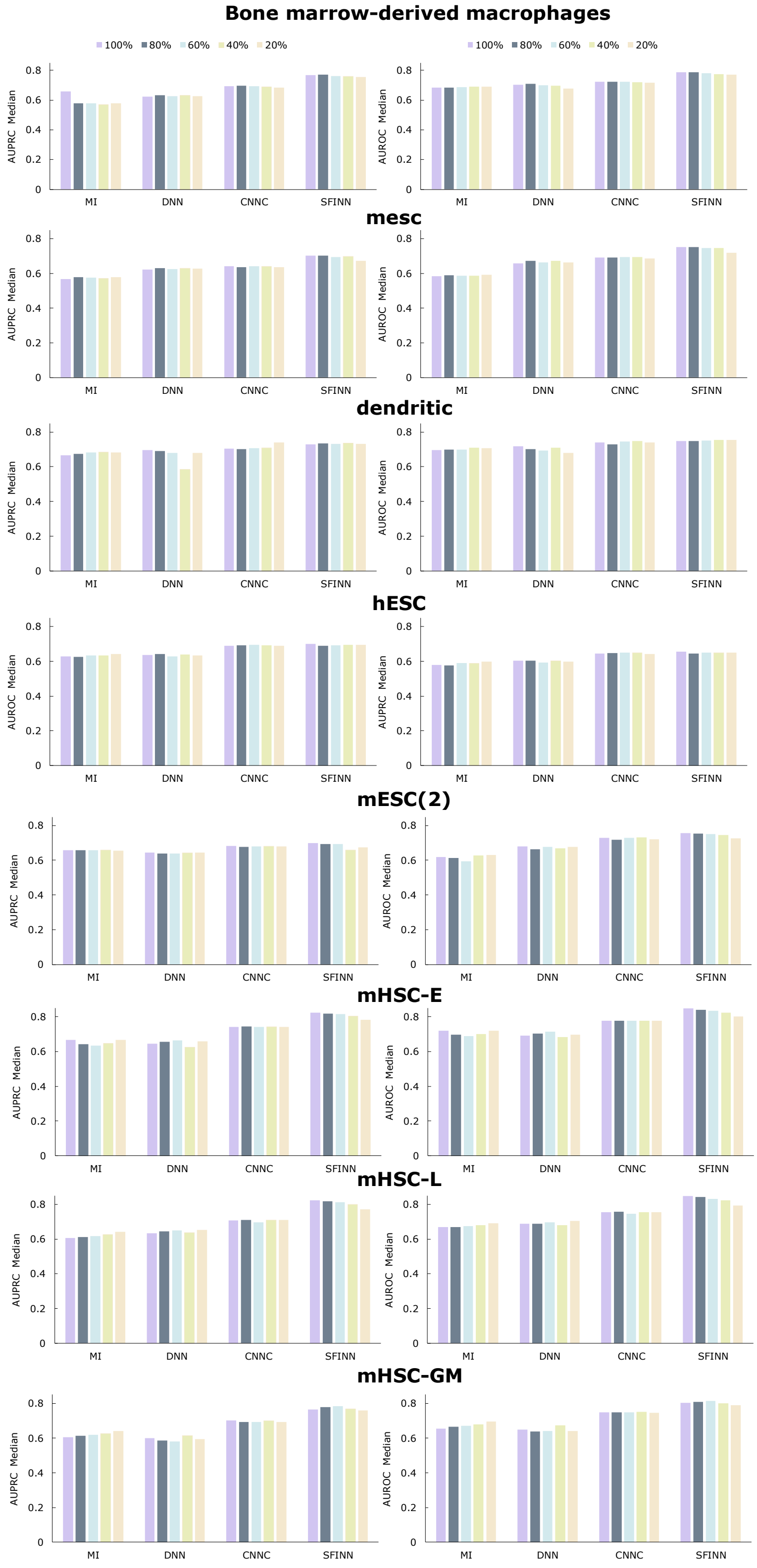

Supplementary Figure 27. The effect of sample size on the performance of SFINN and other compared methods across all single-cell transcriptomic datasets in the task of predicting interactions.

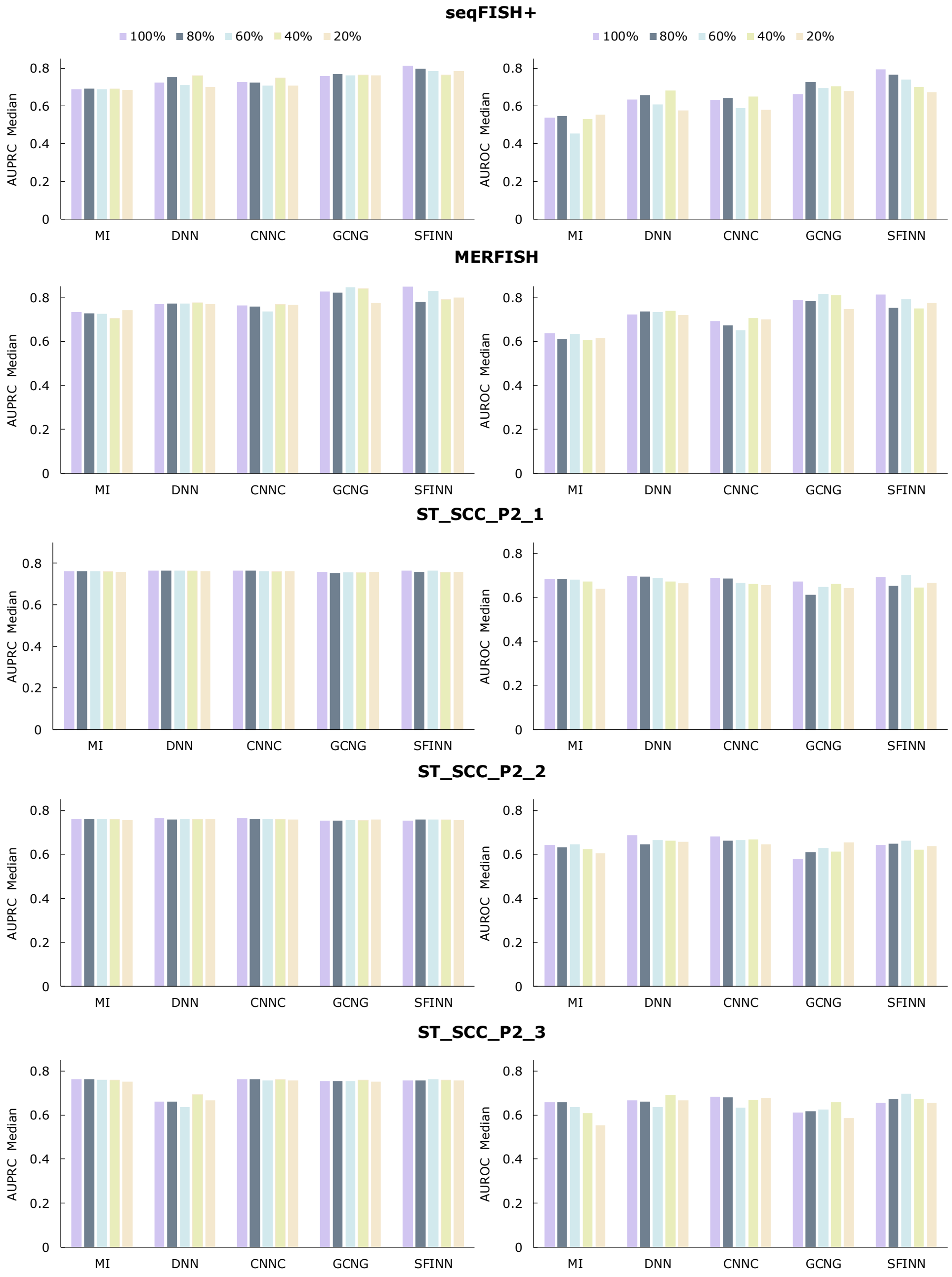

Supplementary Figure 28. The effect of sample size on the performance of SFINN and other compared methods across all spatial transcriptomic datasets in the task of predicting interactions.

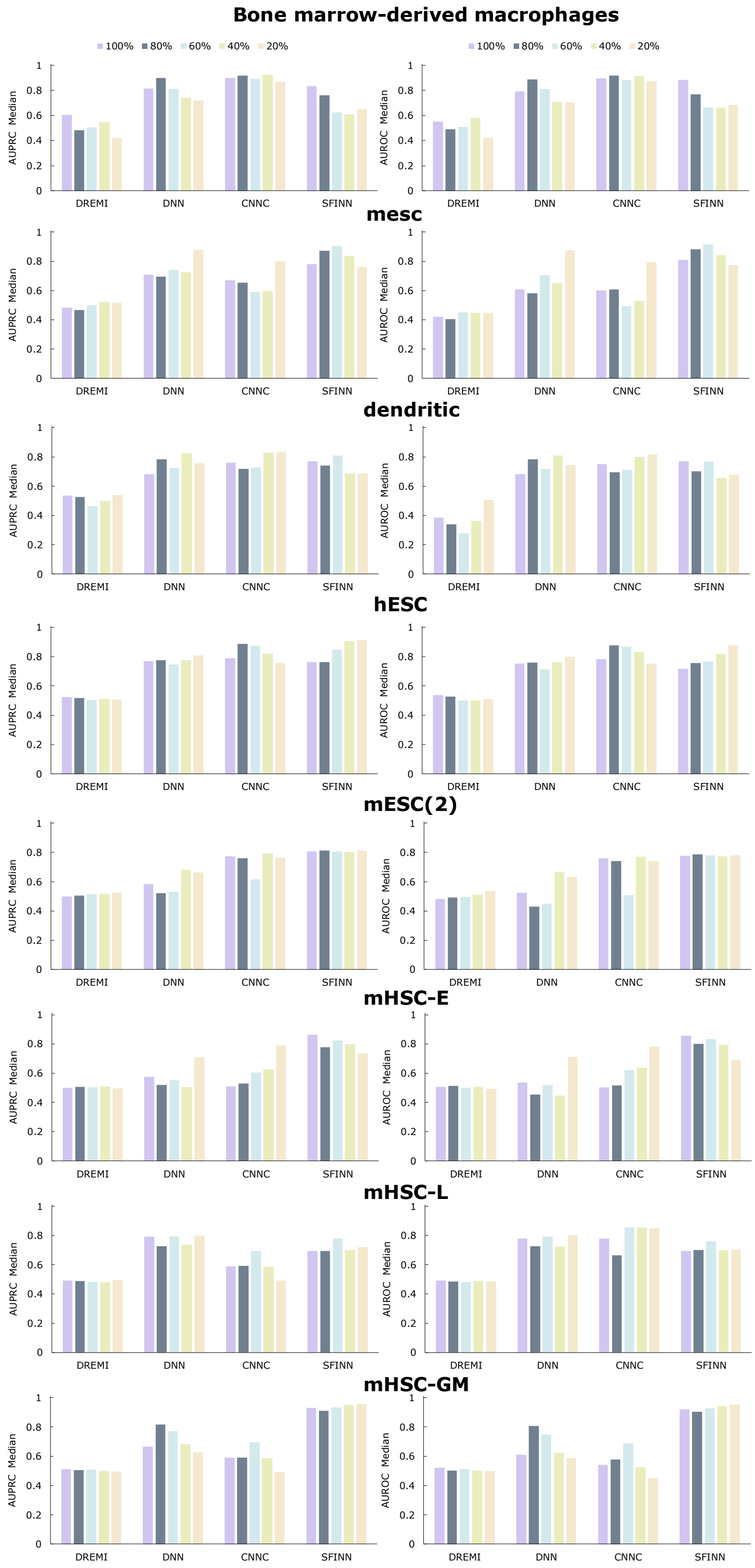

Supplementary Figure 29. The effect of sample size on the performance of SFINN and other compared methods across all single-cell transcriptomic datasets in the task of predicting causalities.

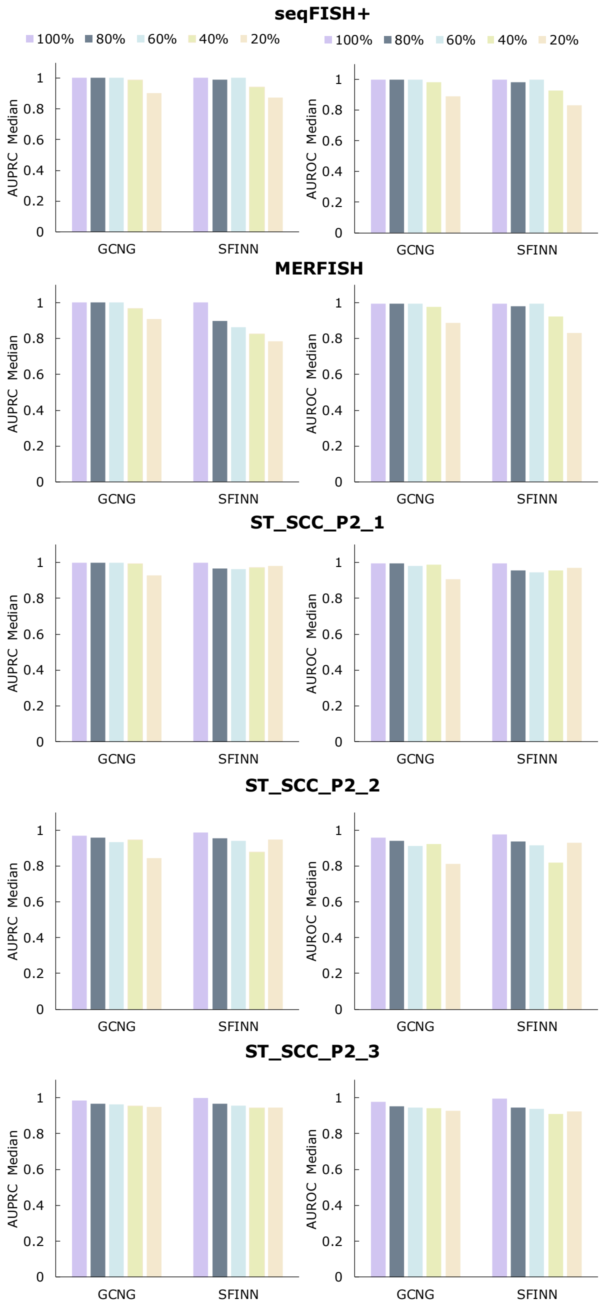

Supplementary Figure 30. The effect of sample size on the performance of SFINN and other compared methods across all spatial transcriptomic datasets in the task of predicting causalities.

A

Top 10 interacting genes for ZNF143 and TBP predicted by SFINN on the hESC dataset

Interacting gene of ZNF143	Score	Ground-truth	Validation	Interacting gene of TBP	Score	Ground-truth	Validation
MIPEP	0.9611	1	Confirmed	MSANTD4	0.9468	1	Confirmed
FAM98B	0.9589	1	Confirmed	MIPEP	0.9455	1	Confirmed
WRAP73	0.9549	1	Confirmed	CEP85	0.9441	1	Confirmed
LPCAT4	0.9540	1	Confirmed	TSC2	0.9415	1	Confirmed
NLE1	0.9538	1	Confirmed	MCM5	0.9401	1	Confirmed
BAIAP2	0.9532	0	Confirmed	CKAP2L	0.9389	0	Confirmed
TSC2	0.9529	1	Confirmed	THOC6	0.9379	1	Confirmed
ARHGEF35	0.9527	1	Unconfirmed	EXOC3	0.9346	0	Confirmed
RRP9	0.9522	1	Confirmed	HIST1H3B	0.9335	1	Confirmed
MSANTD4	0.9514	0	Confirmed	FAM98B	0.9332	1	Confirmed

The interaction network associated with TBP and ZNF143 genes predicted by SFINN

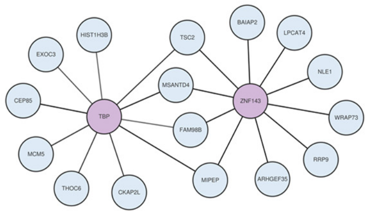

B

Top 10 target genes for ZNF143 and TBP predicted by SFINN on the hESC dataset

Target gene of ZNF143	Score	Ground-truth	Validation	Target gene of TBP	Score	Ground-truth	Validation
C12ORF44	0.9506	1	Unconfirmed	ARHGEF	0.9400	1	Unconfirmed
UBE2T	0.9498	1	Confirmed	FANCE	0.9152	1	Confirmed
MCM5	0.9359	1	Confirmed	C12ORF44	0.9116	1	Unconfirmed
HSPA1B	0.9355	1	Unconfirmed	DDX49	0.9025	1	Confirmed
NSUN5	0.9346	1	Confirmed	LPHN3	0.8849	1	Unconfirmed
AURKB	0.9344	1	Confirmed	TPD52L2	0.8794	1	Confirmed
FANCE	0.9344	1	Confirmed	TCF19	0.8762	1	Confirmed
MARVELD1	0.9317	1	Confirmed	ACTR3B	0.8753	1	Confirmed
HNRNPA0	0.9316	1	Confirmed	HREV1	0.8748	1	Confirmed
MOV10	0.9290	1	Confirmed	FTSJ2	0.8729	1	Confirmed

The regulatory network associated with TBP and ZNF143 genes predicted by SFINN

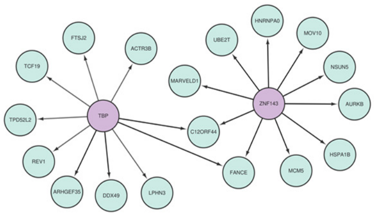

Supplementary Figure 31. The top 10 predictions associated with TBP and ZNF143 genes by SFINN on the hESC dataset for the tasks of (A) gene interaction and (B) causality prediction.

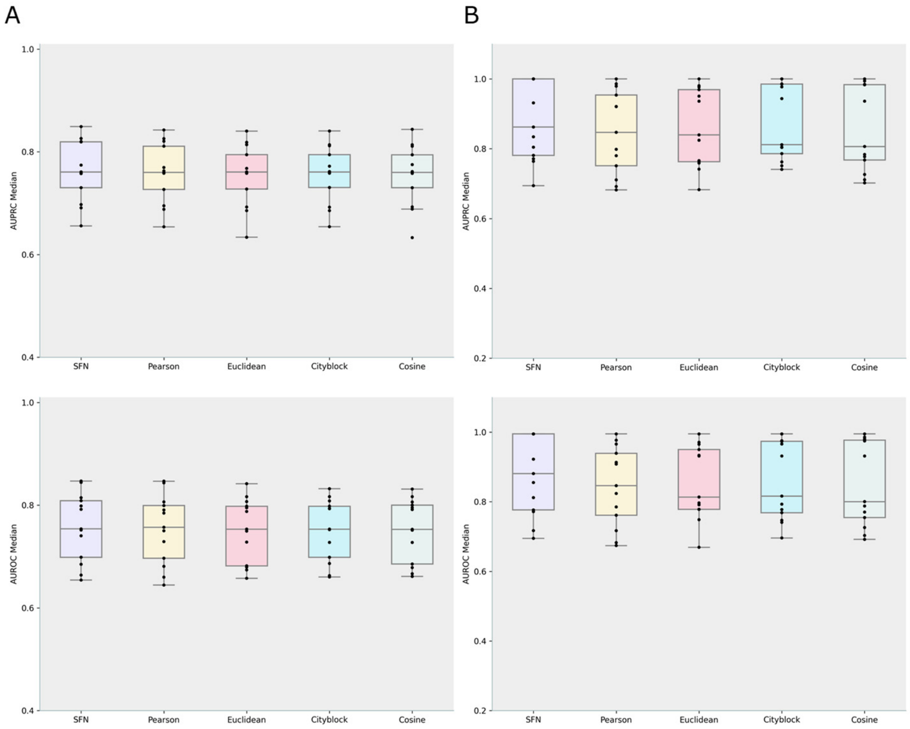

Supplementary Figure 32. The performances across all datasets of model built based on SFN compared with those built based on other commonly used methods for constructing cell-cell network, including Pearson correlation coefficient, Euclidean distance, Manhattan distance, and cosine distance, for (A) TF-gene interaction and (B) causality prediction tasks.
